# Exploring the Effects and Interactions of Conducting Polymers in the Volume Phase Transition of Thermosensitive Conducting Hydrogels

**DOI:** 10.1021/acs.chemmater.4c00433

**Published:** 2024-04-17

**Authors:** David Naranjo, Sofia Paulo-Mirasol, Sonia Lanzalaco, Haoyuan Quan, Elaine Armelin, José García-Torres, Juan Torras

**Affiliations:** † IMEM-BRT Group, Departament d’Enginyeria Química, EEBE, 16767 Universitat Politècnica de Catalunya, C/Eduard Maristany, 10-14, Ed. I, Second Floor, 08019 Barcelona, Spain; ‡ Barcelona Research Center in Multiscale Science and Engineering, EEBE, 16767 Universitat Politècnica de Catalunya, C/Eduard Maristany, 10-14, Basement S-1, 08019 Barcelona, Spain; § Biomaterials, Biomechanics and Tissue Engineering Group, Department of Materials Science and Engineering and Research Center for Biomedical Engineering, Universitat Politècnica de Catalunya (UPC), 08019 Barcelona, Spain

## Abstract

Conducting polymers (CPs) play a vital role in imparting electrochemical and photothermal properties to thermosensitive conducting hydrogels (TCH). The application of TCH is expanding not only for biomedical applications but also to address water scarcity. While the volume phase transition (VPT) phenomenon in thermosensitive polymers has been extensively studied, the contribution of CPs to this process and the underlying chemical interactions remain unclear and low explored. In this study, we present a novel conducting polymer hydrogel (CPH) utilizing the thermosensitive polymer poly­(*N*-isopropylacrylamide) (PNIPAAm) enriched with poly­(3,4-ethylenedioxythiophene) (PEDOT) nanoparticles as a model system. This serves as a platform for both experimental and theoretical investigations into the influence of CPs on VPT. Through a comprehensive examination of hydrogel responses to temperature employing Raman spectroscopy, atomistic simulations using advanced hybrid methodologies, and artificial intelligence, we unveil a shielding effect of CP. This effect arises from robust chemical interactions with NIPAAm, inducing a selective dehydration of the hydrogel microenvironment. Remarkably, this mirrors the phenomenon observed during VPT triggered by an increase in the hydrogel temperature. Understanding the intricate interactions between conducting and thermosensitive polymers is imperative for the systematic development and fine-tuning of the performance of future CPHs. This knowledge ensures a more precise adaptation of these materials to their intended end applications.

## Graphical abstract

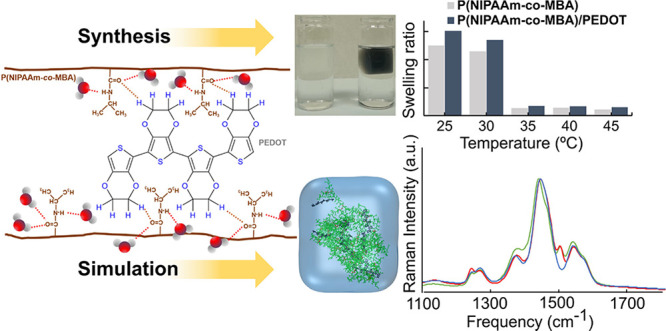

## 1 Introduction

In the past two decades, there has been a growing interest in the exploration and utilization of temperature-responsive polymers.^1–3^ Poly­(*N*-isopropylacrylamide) (PNIPAAm) stands out among them, being a thermoresponsive polymer that undergoes a coil-to-globular conformational transition above its lowest critical solution temperature (LCST) of 305 K.^4^ This transition marks a crucial shift from a highly hydrophilic state (soluble in water) to a hydrophobic condition, causing the polymer to collapse into globular entities that are insoluble in water.^5^

However, the spotlight in recent times has been on the application of PNIPAAm-based hydrogels and their derivatives. The hydrophobic collapse transition in these hydrogels results in a substantial volume change, driving the water expulsion from a fully swollen and hydrophilic hydrogel to one with significantly reduced swelling due to the hydrophobic collapse of its internal structure. This phenomenon is also known as volume phase transition (VPT), wherein hydrogels exhibit the capability to undergo reversible swelling and deswelling on either side of the LCST point.^6^

Thermoresponsive hydrogels have found extensive applications in diverse fields such as biomedicine, energy, coatings, water purification, and more.^7–10^ The integration of the properties of thermoresponsive hydrogels with the characteristics of conducting polymers (CPs) has led to the development of thermoresponsive conducting hydrogels (TCHs).^11,12^ The VPT exhibited by thermoresponsive hydrogels, coupled with the electrochemical and photothermal properties conferred by CPs, renders this composite material highly appealing for potential applications. This innovative approach has garnered significant interest within the scientific community in recent years.^3,12,13^

Given the growing interest in thermosensitive compounds and the fundamental role that PNIPAAm has played as a model system, it is reasonable to assert that the VPT in PNIPAAm hydrogels stands out as one of the most widely studied phenomena in soft condensed matter.^14–17^ Noteworthy among these investigations are studies examining the repercussions on VPT due to different modified PNIPAAm environments. Specifically, the impact on the presence of hydrophobic domains,^18,19^ solvents,^20,21^ and ions and salts,^22,23^ among others, has been thoroughly explored.

One of the most widely employed methodologies for unraveling the intricacies of the microenvironment surrounding PNIPAAm chains during the coil-to-globule transition is the utilization of spectroscopic techniques.^23–26^ Pioneering results, as revealed by FTIR, demonstrated that intermolecular interactions predominantly occurred between water and PNIPAAm below the LCST. Beyond this critical temperature, aggregation ensued due to an escalation in both interchain hydrogen bonding interactions and hydrophobic interactions.^27^ Interestingly, not all hydrogen bonds between PNIPAAm and water are lost in the VPT.^28^ Subsequent Raman studies, in fact, disclosed that only one of the two hydrogen bonds within the carbonyl group is lost, while the hydrogen bond of the amine group remains intact during the hydrophobic collapse.^29^ The role played by water molecules at the atomic level is of high significance. Employing Raman techniques and scrutinizing the intensity relationship of peaks corresponding to the O–H stretching of water molecules allows for the determination of the relationship between water bound to PNIPAAm (strong hydrogen bond) and interstitial water (intermediate strength hydrogen bond).^30,31^ The latter assumes critical importance in the context of TCH applications for water purification through solar evaporation, where understanding the relationship between bound/intermediate/free water elucidates the reduction in evaporation enthalpy.^32,33^

Nevertheless, despite the increasing importance of applications in response to the escalating issue of water scarcity, a knowledge gap exists regarding the chemical interactions between CPs and thermosensitive hydrogels. A deeper understanding of these interactions would enable more accurate fine-tuning of the properties of TCHs when utilized as solar-absorbing hydrogels (SAHs).

In this work, the influence of CPs on the VPT of PNIPAAm will be investigated by using experimental and theoretical approaches. A set of poly­(*N*-isopropylacrylamide-*co*-N,N′-methylenebis­(acrylamide)) (hereafter, P­(NIPAAm-*co*-MBA)) hydrogels will be synthesized for comparison with other samples loaded with nanoparticles of poly­(3,4-etilendioxitiofeno) (PEDOT NPs), denoted as P­(NIPAAm-*co*-MBA)/PEDOT. PNIPAAm and PEDOT materials have been selected as model systems based on their widespread utilization across various fields in recent years. Comprehensive physical-chemical characterization will be conducted, and the hydrogels’ response to temperature will be scrutinized using Raman spectroscopy. The analysis will focus on the blue shift of the prominent 1400–1500 cm^–1^ band during the VPT of the hydrogel across the LCST. Contributions from the deconvoluted sub-bands originating from the deformation vibrations of the −CH_3_ group within the isopropyl moiety of PNIPAAm and the C_α_=C_β_ stretching vibration of the thiophene ring will be taken into consideration. Experimental findings will be validated at the atomistic level through hybrid quantum mechanics/molecular mechanics molecular dynamics (QM/MM MD) simulations. To our knowledge, this represents the pioneering endeavor to simulate a comprehensive 3D network of the PNIPAAm-based hydrogel at the atomistic level, utilizing a broad spectrum of classical, quantum, and hybrid methodologies, alongside its empirical validation. Chemical interactions at the PNIPAAm/PEDOT interface will be scrutinized and a theoretical exploration of the simulated Raman spectrum of the P­(NIPAAm-*co*-MBA/PEDOT) hydrogel bulk will be undertaken, leveraging artificial intelligence, particularly machine learning techniques, for the extraction of pertinent bands from the theoretical Raman spectrum, followed by experimental validation. The use of an artificial intelligence approach aims to enhance efficiency in the subsequent data analysis process. This study endeavors to foster a deeper understanding among researchers regarding the modulation of the VPT of thermosensitive hydrogels through chemical interactions with CPs.

## 2 Materials and Methods

### 2.1 Materials

*N*-isopropylacrylamide (NIPAAm, 97%), *N,N′*-methylenebis­(acrylamide) (MBA, 99%), 3,4-Ethylenedioxythiophene (EDOT, 97%), *N,N,N′,N′*-tetramethyl-ethylenediamine (TEMED, >99%), and dodecyl benzenesulfonic acid (DBSA) were commercially available from Sigma–Aldrich. Ammonium persulfate (APS, >98%) was acquired from Honeywell, Fluka, and Ethanol (99.5%) from PanReac. Milli-Q water grade (0.055 μS cm^–1^) was used in all of the synthesis processes. Unless specified, all chemicals were used as received, without further modifications.

### 2.2 PEDOT Nanoparticles (NPs) Synthesis

The methodology used for the fabrication of PEDOT NPs is very similar to that used in the work of Enshaei et al.^34^ Briefly, 15.8 mL of Milli-Q water and 96 μL of DBSA were added into a 30 mL Corex tube and was stirred at 750 rpm for 1 h in a thermal bath at 40 °C, being protected from light by an aluminum foil. Next, 2 mL of ethanol and 72 μL of the EDOT monomer were slowly added. The mixture was stirred for 1 h at 750 rpm at 40 °C. Finally, 0.73 mg of APS dissolved in 2 mL of Milli-Q water (370 mM) was added drop by drop while stirring. The reaction was left overnight at 40 °C. The side products and unreacted chemicals were removed by a sequence of three centrifugations at 11,000 rpm for 40 min at 4 °C. After each centrifugation, the resulting pellets were dispersed in 15 mL of Milli-Q water with a vortex and a sonication bath (15 min at room temperature). Finally, it was left to dry at 80 °C for 48 h.

### 2.3 PNIPAAm-co-MBA/PEDOT Hydrogel Synthesis

The polymerization of the P­(NIPAAm-*co*-MBA) hydrogel was conducted with a fixed molar ratio (25:1) of NIPAAm and MBA in all the cases. All hybrid conducting polymer hydrogels (P­(NIPAAm-*co*-MBA)/PEDOT), will hold 10% w/w PEDOT NPs. The methodology used is similar to the one previously reported by Lanzalaco et al.^35^ Briefly, 560 mg of NIPAAm, 30 mg of MBA cross-linker, 56 mg of PEDOT NPs, and 8.3 μL of TEMED catalyst were mixed in 20 mL of water in a reaction vessel. The final solution was stirred under a N_2_ gas flow (30 min) to remove dissolved oxygen before the addition of the reaction initiator. Then, 0.15 mL of APS (370 mM) aqueous solution was added to the reaction vessel, allowing the polymerization reaction to proceed for 1 h. A water bath was used to keep the system temperature at 30 °C all the time. Afterward, the resulting hydrogel mixed with PEDOT NPs was extracted and poured onto 400 mL of Milli-Q water. Each sample was maintained under stirring for 4h for purification by continuous replacement of water. Finally, P­(NIPAAm-*co*-MBA)/PEDOT hydrogel samples were stored in a Milli-Q water solution.

The pristine P­(NIPAAm-*co*-MBA) hydrogel was obtained following the same methodology described above but without the addition of PEDOT NPs.

### 2.4 Characterization of Thermosensitive Hydrogel Modified with PEDOT NPs

The morphology of the hydrogel samples was studied by scanning electron microscopy (SEM). Micrographs were acquired in a focused ion beam Neon 40 (Zeiss, Oberkochen, Germany) equipped with an EDX spectroscopy system operating at 5 kV, depending on the sensitivity to beam degradation of the studied systems. The freeze-dried hydrogel was put up on a double-sided carbon disc tape, and a thin layer of carbon was sputter-coated on top to improve the conductivity and prevent charging problems.

Hydrogel porosity was determined by means of microcomputed tomography (micro-CT) analysis using a Skyscan 1272 (Bruker microCT). Micro-CT is an X-ray 3D imaging technique with high resolutions that allows us to obtain layered X-ray images to recover the whole sample in 3D and determine the full porosity of the tested samples. Freeze-dried samples were fixed in a horizontal position to the table inside the device chamber. The measurement was carried out using no filter, a voltage of 50 kV, a power of 10 W, and a current of 200 μA. The angular rotation step was set to 0.2° over an angle of 360°. The resulting voxel size was 3.0 μm for all samples. After the data were obtained, image reconstruction was performed using tNRecon software (Bruker microCT). The final resolution of the method was 0.45 μm. 2D and 3D pictures were reconstructed by using DataViewer and CTVox software.

The swelling ratio of all synthesized hydrogels was conducted at different temperatures. Small samples of the hydrogel were cut, placed in a vial, and frozen at −80 °C overnight. Samples were freeze-dried for 24 h and weighed (*w*_d_). The swelling ratio (SR) was obtained after the immersion of the dry samples in 10 mL of Milli-Q water for 24 h at 25, 30, 35, and 40 °C. The samples were removed from the vials, and the excess water was also removed before weighing the swollen samples (*w*_s_) by capillarity. The swelling ratio was computed according to eq 1.

$$\mathrm{SR}\;(\%)=\frac{w_{s}-w_{d}}{w_{d}}\times 100$$ 1

Fourier transform-infrared spectroscopy (FTIR) analysis was conducted on a Jasco FTIR-4100 spectrophotometer with a resolution of 4 cm^–1^ in the absorbance mode. Freeze-dried samples were placed in an attenuated total reflection accessory with thermal control and a diamond crystal (Golden Gate Heated Single Reflection Diamond ATR). The absorption spectra were obtained after 64 accumulation scans in the region of 600–4000 cm^–1^. Automatic baseline correction was applied using JASCO Spectra Manager software version 2.

The hydrophilic and hydrophobic characteristics of hydrogel systems were assessed through water contact angle (WCA) analysis. WCA measurements were conducted using a KRÜSS Drop Shape Analyzer (DSA25E) at temperatures both above and below the LCST. In each test, a 1 μL water droplet was carefully dispensed onto the sample surface, and this procedure was repeated at five distinct positions on the same sample to ensure accuracy and consistency. Subsequently, the acquired data was processed using the Sessile Drop module within the KRÜSS Advance software suite.

Raman spectra were acquired using a Renishaw dispersive Raman microscope spectrometer (model InVia Qontor, GmbH, Germany) and Renishaw WiRE software, with a spectral resolution of 0.3 cm^–1^ (fwhm), lateral spatial resolution of 0.25 μm and axial spatial resolution <1 μm. To conduct Raman acquisitions at various temperatures, a Linkam accessory (model CCR1000) equipped with a T 96-S heating and freezing stage, connected to a cooling system (Linkam Scientific, Tadworth, UK), was employed. The temperature range was adjusted from 28 to 42 °C, with a controlled heating rate of 1 °C min^–1^. The spectrometer is equipped with a Leica DM2700 M optical microscope, a thermoelectrically cooled charge-coupled device (CCD) detector, and a spectrograph scattered light with 1200 lines mm^–1^ of grating. The experiments were performed at a 785 nm excitation wavelength and with a nominal laser power between 1 and 100 mW output power. The exposure time was 10s, the laser power was adjusted to 1% of its nominal output power and each spectrum was collected with 3 accumulations. All Raman spectra were collected in the spectral range from 1100 to 1800 cm^–1^ with the same measurement parameters.

### 2.5 Quantum Mechanical (QM) Calculations

#### 2.5.1 System Preparation

Ab initio QM calculations were carried out to study the strength of the interactions between the EDOT and NIPAAm molecules. Conformational analyses were performed employing the Gaussian 09 program.^36^ Energy optimization in the gas phase of NIPAAm-EDOT complexes was derived by the Density Functional Theory (DFT) approach using the Minnesota Functionals (M06-2X) in combination with the 6-311+G­(d,p) basis set.^37–39^ A set of NIPAAm-EDOT complexes were built as starting structures to study their conformational and energy stability in the gas phase using preoptimized molecules at the DFT level (Figure S1). More specifically, an EDOT molecule was located in the 26 zones (right rectangular prisms) surrounding the central NIPAAm molecule (considered as a rectangular prism of 11 × 6 × 7 Å). The 26 established zones correspond to the EDOT rectangular cuboid that is in contact with the faces, edges, and vertices of the prism in which the NIPAAm was located. Additionally, different rotation movements were applied to the EDOT residue on the previous structures to yield different relative orientations between both NIPAAm and EDOT residues: (i) the *x*–*y* plane; (ii) 90° counterclockwise rotated around the *x*, *y*, and *z* axes; (iii) 90° clockwise rotated around the *y* and *z* axes; and (iv) 180° anticlockwise rotated around the *y* axis. In total, 26 × 7 rotations = 182 NIPAAm-EDOT complexes were generated to optimize their geometries.

Binding energies were corrected by means of the basis set superposition error (BSSE)^40^ approach applying the standard counterpoise method (CP) as implemented in the Gaussian 09 program.^36^ Consequently, the corrected binding energies (BECP) were computed as detailed in eq 2.

$${\mathrm{BE}}_{\mathrm{CP}}=E_{\mathrm{NIPAM}-\mathrm{EDOT}}-(E_{\mathrm{NIPAM}}+E_{\mathrm{EDOT}})+E_{\mathrm{BSSE}}$$ 2

where *E*_NIPAM–EDOT_ is the QM energy of the optimized complexes; *E*_NIPAM_ and *E*_EDOT_ are the QM energies of EDOT and NIPAAm individually optimized; and *E*_BSSE_ is the energy correction from BSSE approach.

#### 2.5.2 Cluster Analysis

The results from QM calculations were analyzed via Principal Component Analysis (PCA)^41^ and the K-means algorithm^42^ in order to determine clusters of optimized structures based on the geometry and energy of the NIPAAm-EDOT complexes. The geometric and energetic variables were standardized via a standard scaler method, and the number of chosen components was that which described at least 90% of the variance of the total data set. The number of clusters was determined by means of the Elbow Criterion Method which consists of plotting the explained variation as a function of the number of clusters and picking the elbow of the curve as the number of clusters to use.^42^ Cluster analysis was performed employing the scikit-learn library.^43^

#### 2.5.3 Non-Covalent Interactions

Non-Covalent Interactions (NCI) study was performed with the NCIPlot program,^44,45^ which allows the study of the electronic density associated with weak interactions (i.e., van der Waals interactions, hydrogen bonds, and steric clashes), as well as their strength, and their attractive or repulsive nature.

In addition, natural bond orbital (NBO) analysis was performed using the same chemical level as the previous QM calculations (see above), and results obtained from the second-order perturbation theory were also analyzed. NBO aims to provide insight into the bonding interactions between atoms in a molecule. This method is based on a partitioning of the molecular wave function into localized orbitals, called natural bond orbitals, which represent the bonding and nonbonding interactions between atoms in the molecule. On the other hand, second-order perturbation theory analysis involves introducing a perturbation to the system and calculating the resulting change in the electronic energy. This can be used to calculate the second-order correction to the electronic energy and to determine properties, such as vibrational frequencies or infrared spectra.

### 2.6 Molecular Dynamics (MD) Simulations

#### 2.6.1 System Preparation

Two all-atom models of P­(NIPAAm-*co*-MBA) and P­(NIPAAm-*co*-MBA)/PEDOT hydrogels were built employing a Random Search of Energy Minima algorithm implemented in the SuSi (Structure Simulation) program.^46^ The system consisted of 900 residues of NIPAAm and 36 residues of MBA (25:1 molar ratio). First, a sheet of 225 monomers of NIPAAm with 9 MBA moieties was built. In this sheet, 6 anchoring points were defined, as shown in Figure 1a,b, to join 3 additional sheets. The links between the sheets can be summarized as B1—A2, D2—C3, F2—E3, and B3—A4, where the letters represent the anchoring points and the numbers of the sheets (Figure 1c). The final cross-linked-like 3D system is depicted in Figure 1d.

**Figure 1:**
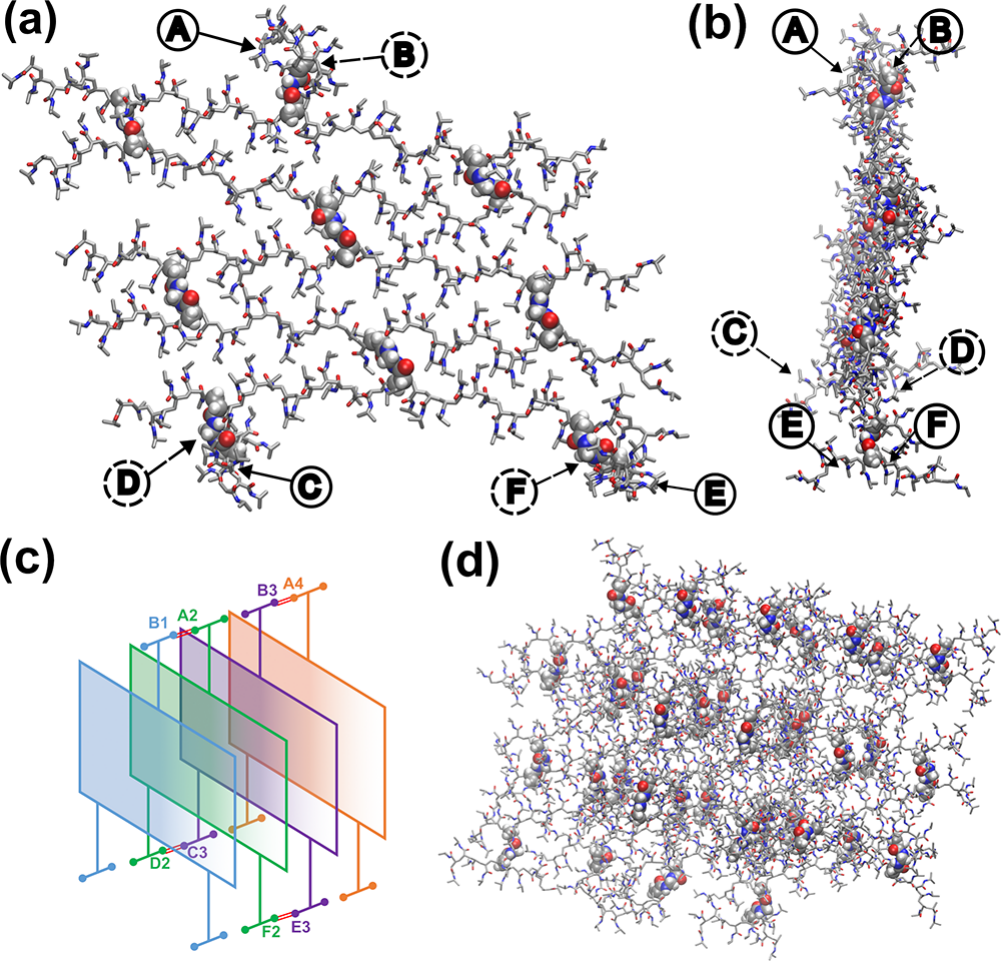
Initial 3D polymer structure of the cross-linked P­(NIPAAm-*co*-MBA) hydrogel system. (a) Front view and (b) side view of one sheet with the six anchoring points. (c) Scheme of the anchoring point distribution among P­(NIPAAm-*co*-MBA) sheets. (d) 3D cross-linked system. NIPAAm monomers are represented in licorice, and MBA atoms (cross-linker molecules) are represented with van der Waals spheres. C atom (gray), N atom (blue), O atom (red), and H atom (white).

The P­(NIPAAm-*co*-MBA)/PEDOT hydrogel systems presents 8 additional units of 8-monomer and one unit of 10-monomer of EDOT, which were randomly added within the P­(NIPAAm-*co*-MBA) structure in order to reach a final 10:1 weight ratio of P­(NIPAAm-*co*-MBA):PEDOT.

The system was parametrized for MD simulations using the LEaP program (AmberTools package), with the General Amber Force Field parameters.^47^ The point charges corresponding to the NIPAAm, MBA, and EDOT residues were derived using the Restrained Electrostatic Potential (RESP).^48^ All simulations were conducted at two different temperatures: one lower than the usual lower critical solution temperature (LCST) of PNIPAAm hydrogels, 25 °C, and another higher than the LCST, 40 °C. Two different solvation boxes were used due to the different behavior on the solvation ratio observed in this type of hydrogel depending on whether it is above or below the LCST point. Thus, in the systems to be simulated at 25 °C, 148403 residues of TIP4P-Ew^49^ water molecules were added in a cubic simulation box with a side of 16.9 nm, whereas in the system at 40 °C, 63110 of TIP4P-Ew water were added to a cubic simulation box with a side of 12.9 nm. Also, in the systems containing doped PEDOT, 37 chloride anions were added to reach electro-neutrality.

#### 2.6.2 Classical Molecular Dynamics Protocol

All MD simulations were conducted using the Amber 18 package.^50^ Initially, the system energy was minimized to remove close contacts and then gradually heated up in the NVT ensemble to the specific simulation temperature (25 or 40 °C) for 60 ps and a time step of 1 fs employing the Langevin thermostat with a collision frequency of 5. Subsequently, a density equilibration stage at 1 atm was run in the NPT ensemble for 0.5 ns and a time step of 2 fs up to constant density. We employed isotropic position scaling using the Berendsen barostat with a relaxation time of 2 ps. The Covalent bonds involving hydrogen atoms were constrained using the SHAKE algorithm.^51^ Finally, the simulation was extended for 400 ns in the NVT ensemble, with coordinates recorded every 20 ps for statistical analysis.

The radius of gyration (*R*_g_) was measured to estimate the spatial extent of the PNIPPAm-MBA system, represented by the mass-weighted root-mean-square distance of all the monomers from the center of the molecule as described in eq 3,

$$R_{g}=\sqrt{\frac{\sum_{i}^{N}m_{i}(r_{i}-r_{C}{)}^{2}}{\sum_{i}^{N}m_{i}}}$$ 3

where *m*_*i*_ is the mass of the *i*th atom and *r*_*i*_–*r*_C_ is the distance to the center of mass.

Moreover, the coordination number, which represents the number of water molecules that are strongly associated with a specific atom through hydrogen bonding or other intermolecular forces, was also measured. More specifically, water oxygen atom and NIPAAm’s oxygen, nitrogen, and methyl carbons were monitored along MD trajectories.

The analysis of hydrogen bonds (HB) networks was conducted using the *cpptraj* module within the AmberTools suite.^52^ HBs are defined as interactions between an acceptor-heavy atom (A), a donor hydrogen atom (H), and a donor-heavy atom (D). An HB is considered formed if the distance between A and D is less than 3.0 Å and the A–H–D angle is greater than 135°. In the identification of potential hydrogen bonds, only hydrogen atoms bonded to oxygen (O) and nitrogen (N) were considered as donors, while O and N were considered as acceptors.

Additionally, the solvent-accessible surface area (SASA) was determined using the *cpptraj* module, wherein solvent-accessible arcs centered around the external atoms of the system were employed to construct the final surface.^53^ The reduction in SASA values, when exposed to a water solvent, provides insights into structural changes associated with the transition from a hydrophilic to a hydrophobic system, offering valuable information about this phenomenon.

### 2.7 Hybrid Quantum Mechanics/Molecular Mechanics-Molecular Dynamics (QM/MM MD) Simulations

#### 2.7.1 System Preparation

P­(NIPAAm-*co*-MBA) systems, previously equilibrated with MD in the presence of excess water, were employed to build smaller systems that accurately represented a solvated and swollen bulk of the hydrogel. Specifically, the simulation unit cell was resized to encompass 95% of the NIPAM, MBA, and PEDOT atoms, thereby excluding any excess water molecules beyond this boundary. Subsequent to resizing, the systems underwent an equilibration stage until they reached a constant density at 1 atm of pressure with a time step of 2 ps. Following MD equilibration, the systems entered a production phase utilizing an NVT ensemble for 40 ns, using system parameters analogous to those used in the previously described MD simulations. Once the trajectories reached a steady state with a constant RMSD, 10 snapshots were extracted from each system for an ulterior simulation at the QM/MM MD level.

#### 2.7.2 QM/MM MD Protocol and Raman Frequency Calculation

The QM/MM calculations were conducted using the PUPIL program,^54,55^ facilitating QM/MM coupling while utilizing the Gaussian program for calculations at the QM level and Amber suite for classical MD simulations. In P­(NIPAAm-*co*-MBA) systems, the QM zone was composed of a single MBA residue linked to one NIPAAm residue. For P­(NIPAAm-*co*-MBA)/PEDOT systems, the QM zone involved two connected EDOT residues (dimer) surrounded by three NIPAAm residues, totaling three amide groups within the QM region. The PBE1PBE/6-31G­(d,p) level of theory was applied for all QM calculations, incorporating a 15 Å cutoff radius for electrostatic interactions around the QM region, considering classical atoms as point charges. Ewald sums were considered for the long-range interactions.

In each of the ten snapshots from the simulated systems (P­(NIPAAm-*co*-MBA) and P­(NIPAAm-*co*-MBA)/PEDOT at 25 and 40 °C), an initial minimization was performed at the QM/MM MD level using 200 steps of the steepest descent algorithm followed by conjugate gradient algorithm up to 500 steps in total. Subsequently, Raman frequency calculations for the quantum zone were conducted at the theoretical level of PBE1PBE/6-31G­(d,p). Considering all the frequency spectra obtained, the normal modes of amide I, amide II, amide III, CH_3_ deformation of the isopropyl group of PNIPAAm, and the stretching vibration of C_α_=C_β_ in the five-membered ring of EDOT residues, were labeled. Based on the labeled spectra, two artificial neural networks (ANNs) were then constructed and trained for the automatic identification of normal vibration modes.

The first ANN used the frequency, infrared intensity, Raman activity, and displacement vectors of the amide groups with the O, N, H, and C atoms as input variables. The second ANN utilized frequency, infrared intensity, Raman activity, displacement vectors of the C atom, and the six H atoms of the isopropyl group of NIPAAm, along with stretching vibration C_α_=C_β_ in the five-membered ring of the EDOT residue.

Out of the 40 snapshots (10 per system), 70% were allocated to the training set, 15% were allocated to validation, and the remaining 15% for testing. The first ANN had an architecture of 7–100–100–100–4 (7 neurons in the input layer, followed by three hidden layers with 100 neurons each and an output layer with 4 neurons), and the second ANN had an architecture of 13–100–100–100–100–3. These ANNs were implemented using the Keras library with the TensorFlow backend,^56^ configuring layers with the *Keras* sequential model using the *ReLu* activation function in hidden layers and *softmax* activation function in the outer layer. The Adam optimizer was chosen to minimize the categorical cross-entropy loss function.^56^

Evaluation of the validation set ensured model performance and prevented overfitting, employing metrics such as accuracy, precision, recall, and the F1 score (harmonic mean of precision and recall). Subsequently, a QM/MM MD simulation of 6 ps under an NVT ensemble was performed on the last snapshot of each system. Once the RMSD reached a plateau in the resulting trajectories, 25 snapshots were extracted for each system. Each snapshot underwent QM/MM MD minimization and frequency calculations in the QM zone. The pretrained ANN models were then applied to quickly extract information from all spectra obtained for subsequent analysis of the normal modes in the amide, isopropyl of NIPAAm, and the thiophene ring.

## 3 Results and Discussion

### 3.1 Hydrogel Fabrication

In the design of new thermosensitive conductive hydrogel materials, it is of crucial importance to understand the change in chemical interactions between the thermosensitive hydrogel and the supported conductive materials along the LCST point.^33,57^ Thus, in this study, we synthesized a soft conductive polymer hydrogel by copolymerizing NIPAAm with MBA and loading it with PEDOT NPs, denoted as P­(NIPAAm-*co*-MBA)/PEDOT. This hydrogel was designed to exhibit excellent electrochemical properties. The resulting conductive hydrogel served as an effective experimental platform for comparing and validating simulated data related to chemical interactions occurring around the LCST point.

Throughout the synthesis of P­(NIPAAm-*co*-MBA) hydrogels, a constant molar ratio of 25:1 between NIPAAm and MBA was maintained. Additionally, the weight ratio of PEDOT NPs to P­(NIPAAm-*co*-MBA) was fixed at 10:1 in the final formulation through preliminary experimentation, aimed at preserving the mechanical integrity within the hydrogel matrix. The delicate equilibrium between the PEDOT content and the hydrogel’s mechanical strength is intricately influenced by its alginate composition. The hydrogels were fabricated through free radical polymerization (Figure S2), utilizing APS as the radical initiator. PEDOT NPs were synthesized via emulsion polymerization with APS as the oxidative agent. After NP isolation, they were added to the reaction vessel along with other reactants before the NIPAAm polymerization. The resulting NPs had an average size of 106 ± 20 nm as can be seen in Figure S3 (Supporting Information).

### 3.2 Hydrogel Characterization

FTIR analyses were performed to characterize PEDOT NPs and P­(NIPAAm-*co*-MBA) and P­(NIPAAm-*co*-MBA)/PEDOT hydrogels (Figure 2a). PEDOT NPs show the characteristic FTIR peaks of PEDOT chains, which appear at 1635 and 1473 cm^–1^ (C=C stretching), 1344 cm^–1^ (C–C stretching), 1220 and 1061 cm^–1^ (C–O–C vibrations) and 842 cm^–1^ (stretch of the C–S bond in the thiophene ring). Also, weak but clearly defined bands attributed to DBSA dopant molecules were detected at 2920 and 2850 cm^–1^ (−CH_2_ and–CH_3_ stretching), and 1693 cm^–1^ (C=C stretching from the phenyl group).^58^ The FTIR spectrum of PNIPAAm (Figure 2a) shows the absorption bands of the characteristic amide at 1640 cm^–1^ (C=O stretching of amide I), 1530 cm^–1^ (N–H bending of amide II), and 1170 cm^–1^ (C–N of amide III). The absorption bands at 1459 cm^–1^ were attributed to C–H bending in −C­(CH_3_)_2_ and −CH_2_– groups. The absorption region below 3000 cm^–1^ corresponded to C–H stretching vibrations of −CH_2_– groups. The peaks at 2965 and 2942 cm^–1^ belong to CH_2_ and CH_3_ stretching, respectively. Finally, the peaks at 3290 and 3436 cm^–1^ are characteristic bands of the valence vibration N–H and O–H groups, respectively.^59^

**Figure 2:**
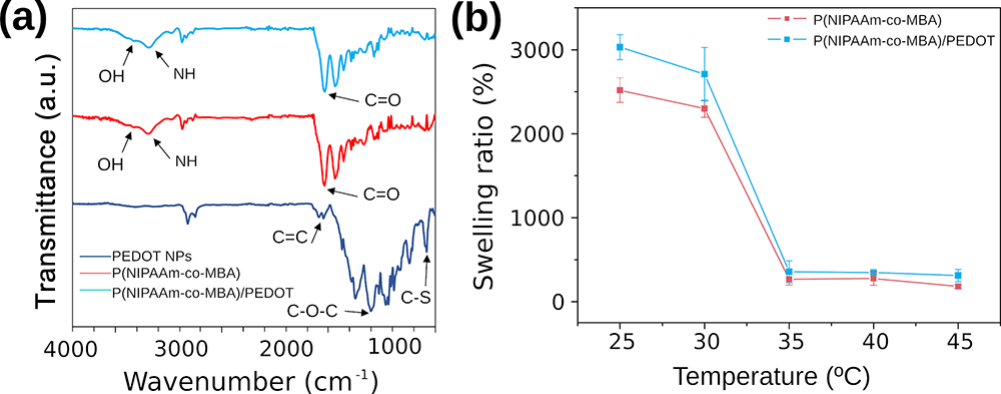
(a) FTIR spectra of PEDOT NPs (dark blue line), P­(NIPAAm-*co*-MBA) (medium blue line) and P­(NIPAAm-*co*-MBA)/PEDOT (light blue line). (b) Swelling ratio of P­(NIPAAm-*co*-MBA) and P­(NIPAAm-*co*-MBA)/PEDOT at different temperatures.

The strong variation in the SR that PNIPAAm hydrogels experiment with a small temperature change around the LCST region (about 32 °C) is well known.^60^ Upon crossing the LCST point, the hydrogel undergoes an important VPT that affects the SR. In order to determine the effect produced by the presence of PEDOT NPs, the SR was measured and calculated using eq 1 at different temperatures of 25, 30, 35, and 40 °C. As can be seen in Figure 2b, P­(NIPAAm-*co*-MBA)/PEDOT presents a higher SR (3031%) compared with bared P­(NIPAAm-*co*-MBA) (2517%) at 25 °C. Above the LCST point, both hydrogels presented a similar SR. The difference in the SR obtained at 25 °C in both systems suggested that the addition of PEDOT NPs to the 3D network of the hydrogel has a direct influence on the cross-linking process and the microporous structure, leading to higher porosity and a significant increase in SR.

The morphologies of pristine P­(NIPAAm-*co*-MBA) and P­(NIPAAm-*co*-MBA)/PEDOT hydrogels were characterized by SEM and the volumetric porosity was approached by micro-CT. In Figure 3a, SEM images depict the hydrogels at various magnifications, showcasing their interconnected open-pore structure, which helps promote water absorption and transport through the 3D network of the hydrogel. Figure 3b zooms in on the microporous surface of P­(NIPAAm-*co*-MBA)/PEDOT, revealing the presence of PEDOT NPs which enhance hydrophobicity and promote increased porosity. The impact of CP on the pore size is evident in the micro-CT analysis depicted in Figure 3c. The pristine hydrogel showed an average pore size of 12 μm, while that of P­(NIPAAm-*co*-MBA)/PEDOT was 24 μm. Indeed, the total porosity obtained was 48.3 and 79.4% for the P­(NIPAAm-*co*-MBA) and P­(NIPAAm-*co*-MBA)/PEDOT hydrogels, respectively.

**Figure 3:**
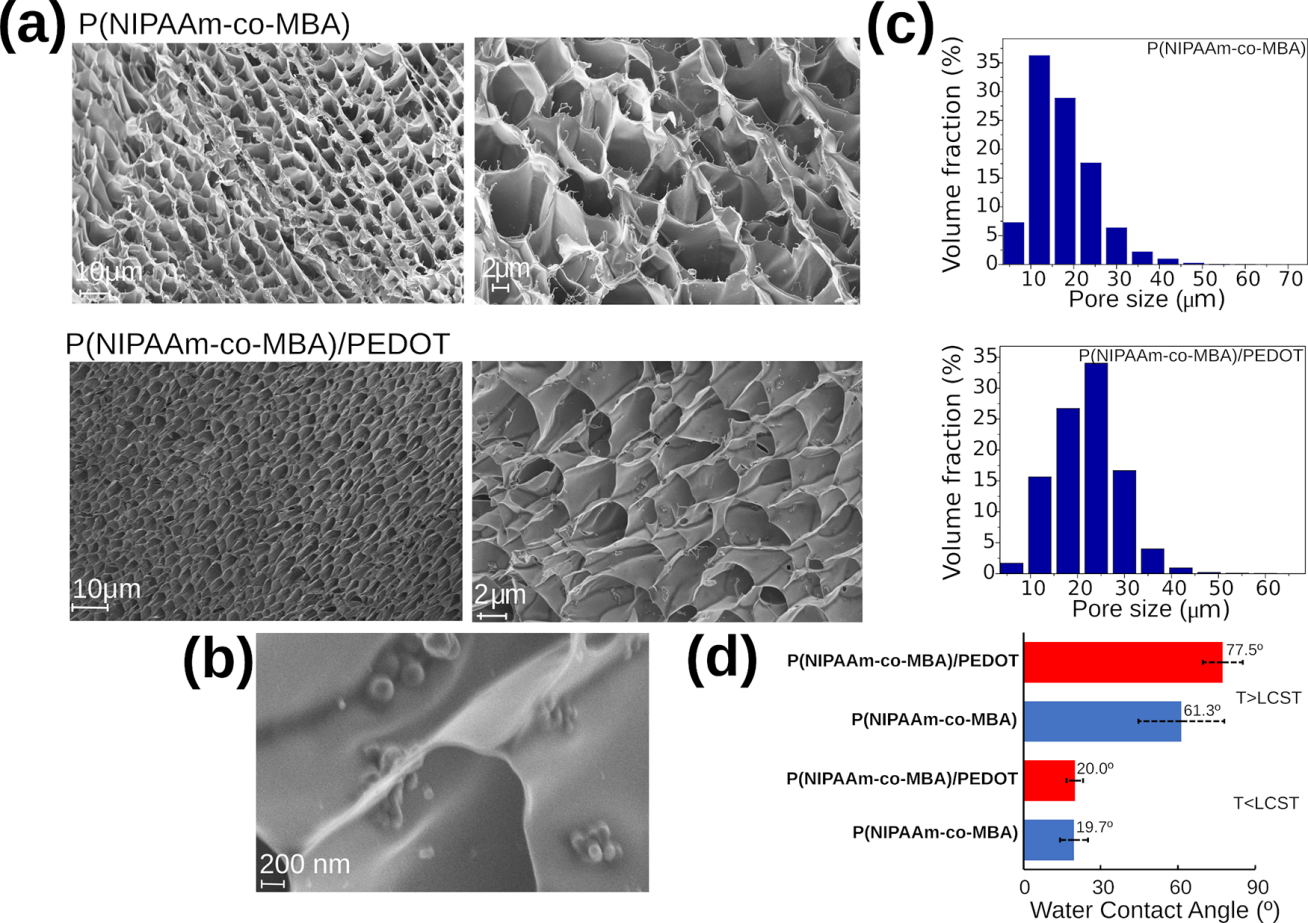
(a) SEM images showing the cellular structure and well-distributed porous networking formation in pure (top) and hybrid hydrogel (down). (b) Detail of the PEDOT NPs deposited on the surface of the hydrogel. (c) Porosity distribution of P­(NIPAAm-*co*-MBA) and P­(NIPAAm-*co*-MBA)/PEDOT hydrogels determined by micro-CT. (d) Water contact angle of both hydrogels at the pre- and post-LCST points.

The hydrogel loaded with PEDOT NPs almost doubled the porosity of its pristine form, which corroborates the large influence of PEDOT NPs on the cross-linking process. In addition, we examined the influence of CPs on the hydrophilic–hydrophobic transition along the VPT. Water contact angle (WCA) measurements were conducted before and after the LCST on both hydrogels: the pristine one and the variant loaded with PEDOT NPs. As depicted in Figure 3d, there is a notable increase in hydrophobicity along the VPT due to the presence of CPs. Prior to the LCST, the difference in hydrophilicity was minimal (ΔWCA = 0.3°). However, post-VPT, the WCA triples (61.3° ± 16.7°) for the P­(NIPAAm-*co*-MBA) hydrogel and nearly quadruples (77.5° ± 7.8°) for the P­(NIPAAm-*co*-MBA)/PEDOT variant. Clearly, the CPs significantly enhance the hydrophobicity above the LCST.

### 3.3 Evaluation of the Temperature Effect by Raman Spectroscopy

The conformational change in the P­(NIPAAm-*co*-MBA)/PEDOT samples at different temperatures was further studied by Raman spectroscopy. The swollen and collapsed states of PNIPAAm accuse changes in the hydrogen bonding interactions inside the polymer chain.^3^ Therefore, independent of the application envisaged for such systems, small variabilities of external temperature will induce big changes in either the intramolecular and intermolecular interactions of NIPAAm with other molecules. The Raman spectra of P­(NIPAAm-*co*-MBA) and P­(NIPAAm-*co*-MBA)/PEDOT samples, depicted in Figure 4, were analyzed at temperatures below, near, and above the LCST transition temperature. These temperatures were precisely controlled by using a Linkam accessory.

**Figure 4:**
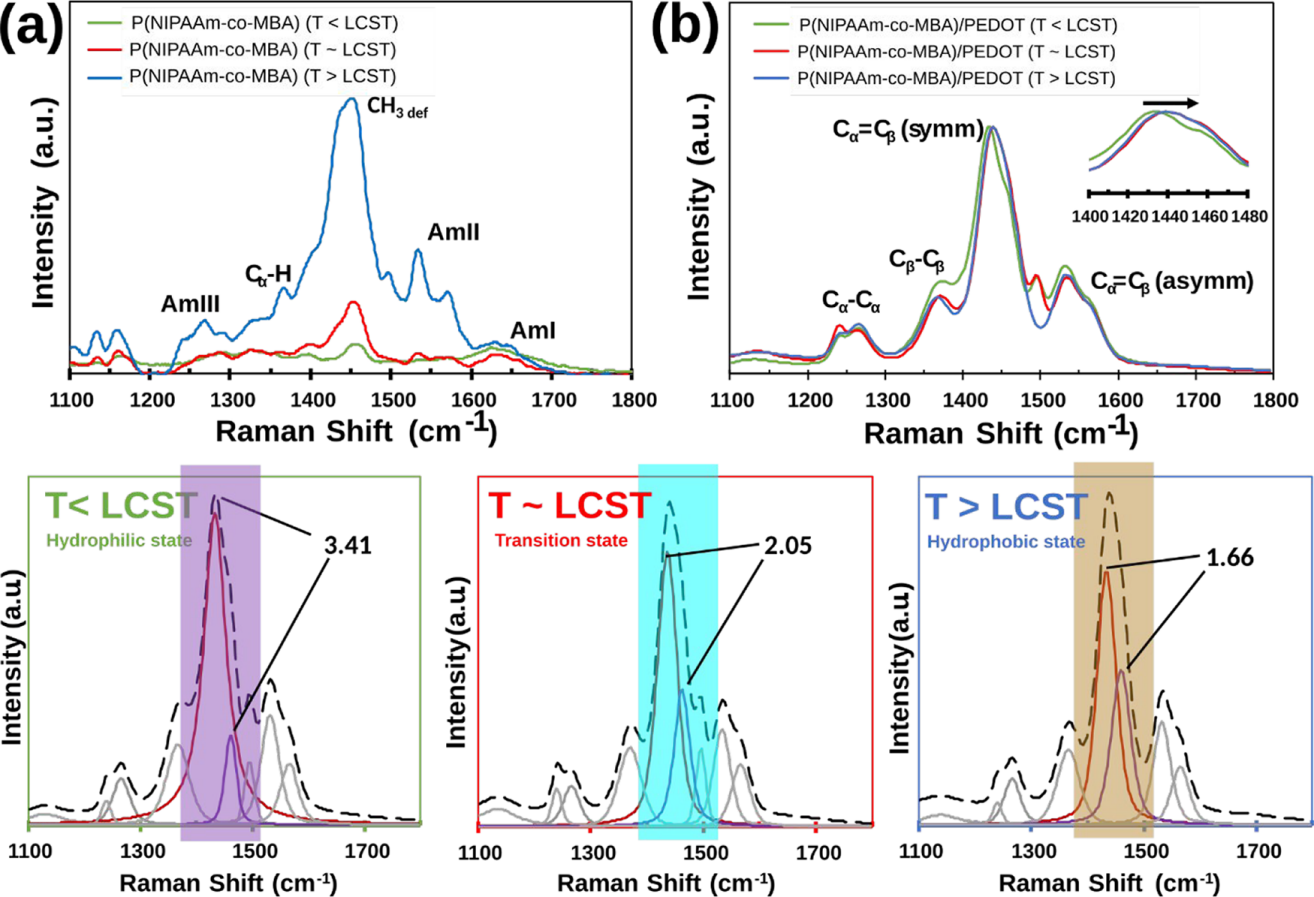
Raman spectra of P­(NIPAAm-*co*-MBA) (a) and P­(NIPAAm-*co*-MBA)/PEDOT (b) at different temperature across the LCST. Deconvolution of spectra recorded at *T* < LCST, *T* ∼ LCST and *T* > LCST for P­(NIPAAm-*co*-MBA)/PEDOT samples.

The core of this study is the understanding of the intra-intermolecular arrangement of the thermosensitive hydrogel chains under temperature stimulus and in the presence of conductive polymer NPs. The shrinking of PNIPAAm-based gels across the LCST has been reported to involve molecular changes that can be probed by Raman spectroscopy.^61^ We have also noted the switch of certain absorption bands in another study.^62^ For instance, the intensity of the CH_3_ stretching mode of the methylene group is often correlated to the lateral packing density of the polymer chains. Any shifts or changes in intensities of other absorption bands can be exploited to study the polymer/polymer and polymer/solvent interactions. Herein, we focused our attention on the CH_3_ and C=O stretching regions of the PNIPAAm in the range between 1100 and 1800 cm^–1^, with these functional groups being responsible for their hydrophobic and hydrophilic states.

As observed in Figure 4a, five main important bands have been identified in this range, the C=O stretching region of the amide groups as amide I (AmI), II (AmII), and III (AmIII), pointed at 1628, 1558, and 1255 cm^–1^, respectively. In addition, the C_α_H stretching mode (1389 cm^–1^) and CH_3_ deformation (1460 cm^–1^, hereafter ν_defCH3_) of the isopropyl group of PNIPAAm were also verified.^63^ During the heating process, from lower to higher temperatures than that of LCST, an increase in the peak intensity was observed for PNIPAAm-*co*-MBA samples, suggesting that the coil-to-globule transition of PNIPAAm induces an increase in the packing density of the polymer chains in the shrunken state. Figure 4b displays the temperature-dependent Raman spectra of PNIPAAm-*co*-MBA loaded with PEDOT NPs and dispersed inside the gel matrix. The most obvious change was observed for the strongest band between 1400 and 1500 cm^–1^, where the conductive PEDOT NPs exhibited a narrower and intense band located at 1435 cm^–1^. The last vibrational mode corresponds to the symmetric stretching vibration of C_α_=C_β_ (hereafter, ν_stCα=Cβ_) on the five*-*member ring of PEDOT.^64^ This band demonstrates a correlation with the resonant structure of PEDOT, exhibiting a shift toward either the red or the blue spectrum depending on the type of doping applied. This phenomenon gives rise to quinoid and benzoid structures for the linear and coil conformations, respectively.^65,66^ Other bands have been individuated in the same Raman shift interval, the C_α_–C_α_ inter-ring and the C_β_–C_β_ stretching modes located at 1255 and 1368 cm^–1^, respectively, as well the peak located at 1508 cm^–1^, associated with the C_α_=C_β_ asymmetric stretching vibrations.^67^ Focusing the attention onto the ν_stCα=Cβ_ peak, a blue shift of ∼20 cm^–1^ (Δ cm^–1^) has been observed with increasing temperature (inset of Figure 4b). It reflects the phase transition temperatures of PNIPAAm-*co*-MBA/PEDOT hydrogels during the heating process, from the hydrated to the dry forms. The deconvolution of the entire Raman spectra recorded in the presence of PEDOT NPs has been carried out at three different temperature ranges (*T* < LCST, *T* ∼ LCST, and *T* > LCST of Figure 4). The deconvoluted peaks perfectly match those previously commented for both pristine polymers (i.e., PNIPAAm and PEDOT). The effect of the temperature on the conformational changes has been observed by measuring the ratio (ϕ = *I* ^Ram^_stCα=Cβ_/*I* ^Ram^_defCH3_) between the Raman-scattering activity of the peaks centered at 1435 cm^–1^ (ν_stCα=Cβ_) and 1460 cm^–1^ (ν_defCH3_). This ratio decreases from 3.41 to 1.66 from hydrophilic to hydrophobic states due to the intensity increase of the ν_defCH3_ peak of PNIPAAm (Figure 4a). Therefore, the reorganization of the neighboring water molecules leads to the dehydration of the isopropyl group in both PNIPAAm-*co*-MBA and PNIPAAm-*co*-MBA/PEDOT hydrogels. The temperature behavior of the CH_3_ frequency confirms that the main features of the coil-to-globule transition of PNIPAAm are preserved, although the reduced hydration of the isopropyl group of NIPAAm is accompanied by a new mechanism, most probably ascribable to the steric hindrance of PEDOT NPs, limiting the hydrogel shrinking. The combined effect of reduced hydration of the isopropyl group of PNIPAAm and of the topological rearrangements of the polymer networks will be following elucidated by computational simulation studies.

### 3.4 NIPAM/PEDOT Interactions

The investigation of the modulation in the hydrophilic–hydrophobic state transition of PNIPAAm, influenced by the presence of a conducting polymer, requires a comprehensive examination across various levels of approximation. This entails a deep insight into the chemical interactions at the atomistic level within the diverse monomers of both polymers, exploring topological arrangements among polymeric networks and scrutinizing molecular interactions among polymer chains and their interactions with surrounding water.

To initiate this exploration, an in-depth study was conducted on the interactions within the complex formed by monomeric residues of both polymers (i.e., NIPAAm-EDOT) at the QM level. By arranging both monomers in various positions and orientations in space with the NIPAAm molecule at the center and the EDOT molecule in the surrounding spatial sphere, 182 initial structures of the NIPAAm-EDOT complexes were meticulously examined. Optimized geometries and binding energies (BE) were obtained for each structure, accounting for base superposition effects, as detailed in the methods section.

All optimized structures at the QM level underwent processing using principal component analysis (PCA)^41^ and the K-means algorithm^42^ to identify contrasting structures based on geometry and the final binding energy of NIPAAm-EDOT complexes. The PCA results revealed that a remarkable 98.6% of the data set’s variance could be explained with just three principal components (PC). These PCs were selected as the new variables to establish the total number of clusters for the subdivision of all optimized complexes. The evolution of the summation of squared distances of the PCs (comprising geometric and energetic variables) against the total number of clusters is depicted in Figure S4, indicating that the optimal number of clusters for describing all of the optimized NIPAAm-EDOT complexes set is 15. Table S1 provides the BE among the 15 clusters of NIPAAm-EDOT complexes and the distances between the centers of mass of each complex component. The top four most stable complexes within this selection exhibit a binding energy range of −6.52 to −8.26 kcal mol^–1^ and the shortest distances, ranging from 3.6 to 4.8 Å, between the centers of mass of each component within the complex.

Figure 5 displays optimized geometries alongside noncovalent interactions (NCI) isosurfaces for the four most stable clusters. Blue and green colors represent attractive weak interactions, such as hydrogen bonds and extremely weak interactions (van der Waals), respectively. Red color indicates repulsive interactions, such as steric clashes. The NCI study reveals that with the exception of the last three clusters, all systems exhibit a predominant van der Waals interaction. Notably, the more stable cluster complexes (clusters A–D) feature a C=O···H interaction, observed between the C=O moiety of the NIPAM molecule and a hydrogen atom on the dioxane ring of EDOT. The O···H distances for representatives of clusters A (Figure S5), B, C, and D are 2.22, 2.40, 2.91, and 3.21 Å, respectively.

**Figure 5:**
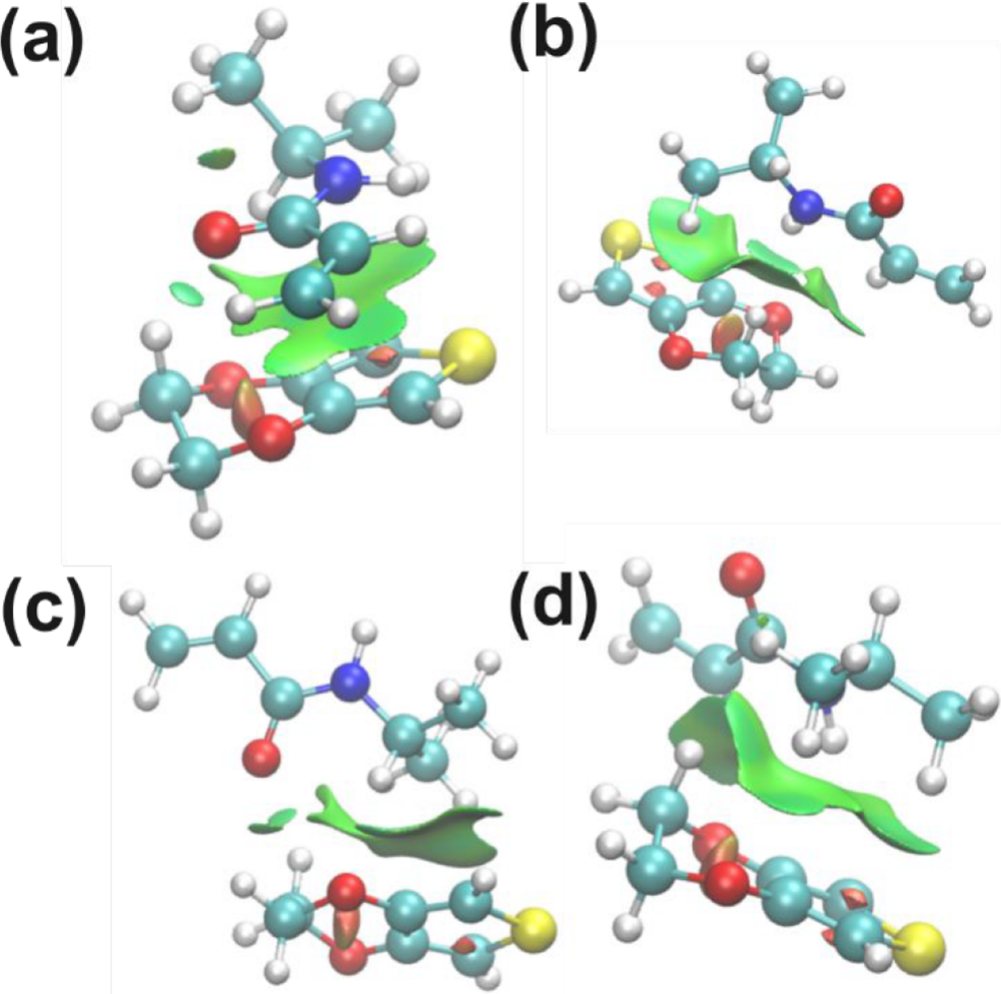
Optimized geometries of NIPAM-EDOT complexes and NCI isosurfaces for representative molecules of clusters A, B, C and D.

Furthermore, NBO analysis supplements this understanding by providing information about the strength and nature of the orbital interactions. When coupled with second-order perturbation theory, NBO analysis enables the identification and quantification of interactions between atoms, offering deeper insights into the chemical bond nature.^68^ In the most stable NIPAAm-EDOT complex (cluster A), second-order perturbation theory analysis reveals that the interaction between the lone pair of oxygen atoms in the carbonyl moiety and the hydrogen atom of the dioxane ring in EDOT contributes to the most significant stabilization energy, amounting to 1.54 kcal/mol. Interestingly, none of the studied clusters exhibit a substantial N–H···O interaction between the amino group of the NIPAM monomer and the oxygen atom in the dioxane ring of the EDOT.

### 3.5 Temperature Effect of the P­(NIPAAm-*co*-MBA)/PEDOT Hydrogel

MD simulations were conducted on two atomistic systems, namely, P­(NIPAAm-*co*-MBA) and P­(NIPAAm-*co*-MBA)/PEDOT, both solvated in excess water. The primary focus was to investigate the hydrophilic–hydrophobic state transition at the LCST point. Two distinct simulation temperatures were selected to represent the pre- and post-LCST states, enabling a clearer observation of changes in interactions at the interface between the hydrogel and the conductive polymer. A summary of the averaged values of observable variables from the last 200 ns of the MD trajectory is presented in Table 1, showcasing data at two temperatures: pre-LCST (25 °C) and post-LCST (40 °C). The experimentally determined LCST point for our systems is approximately ∼32.5 °C, as illustrated in Figure 2b. An evident decrease in the SR is observed in both systems, consistent with the hydrophilic–hydrophobic transition of PNIPAAm reported elsewhere.^5^ This state transition, at the atomistic level, is primarily characterized by alterations in volume and interactions with the solvent.^6^

**Table 1:** Average Values on the Radius of Gyration (RoG, in Å), Solvent Accessible Surface Area (SASA, in Å^2^), Number of Solute–Solute Hydrogen Bonds (UU-HBs), and Number of Solute–Solvent Hydrogen Bonds (UV-HBs) of P­(NIPAAm-*co*-MBA) and P­(NIPAAm-*co*-MBA)/PEDOT at 25 and 40 °C^a^

	P(NIPAAm-*co*-MBA)		P(NIPAAm-*co*-MBA)/PEDOT	
	25 °C	40 °C	25 °C	40 °C
RoG	34.9 ± 0.1	33.0 ± 0.0	39.9 ± 0.1	36.4 ± 0.0
SASA	70108 ± 160	66046 ± 43	74853 ± 112	73051 ± 49
UU-HBs	35 ± 0	47 ± 0	34 ± 0	47 ± 0
UV-HBs	1708 ± 3	1545 ± 7	1750 ± 4	1601 ± 1

a All measurements were derived from MD trajectories. Standard deviation is also shown.

The observed change in the radius of gyration (RoG, Table 1 and Figure S6a) for both systems aligns qualitatively with experimental results, displaying a decrease of 5.4 and 8.8% for the P­(NIPAAm-*co*-MBA) and P­(NIPAAm-*co*-MBA)/PEDOT systems, respectively. Indeed, upon surpassing the LCST, PNIPAAm is anticipated to adopt a globular conformation, leading to a smaller radius of gyration at the atomistic level. Previous MD simulations have consistently reported this enhanced volume reduction, attributing it to the hydrophobic collapse of the 3D network within the PNIPAAm hydrogel.^69–72^ Intriguingly, the system loaded with CP demonstrated a more pronounced volume reduction compared to that of the pristine system. The observed VPT phenomenon appears to be amplified by the presence of CP, aligning seamlessly with experimental results as depicted in Figure 2b. However, in the P­(NIPAAm-*co*-MBA)/PEDOT system, the RoG is larger than that of the system without PEDOT at both temperatures. This discrepancy with the experimental data can be attributed to the expanded system size resulting from the introduction of additional PEDOT chains within the simulated bulk and the hydrogel macro- and microporosity that is not considered in the current hydrogel bulk simulation at the atomistic level. A parallel trend is observed in the solvent-accessible surface area (SASA) results listed in Table 1 (Figure S6b).

An interesting aspect to explore at the atomistic level involves examining changes in the network of solute–solute (UU) and solute–solvent (UV) hydrogen bonds, as detailed in Table 1 and visually depicted in Figure S6c,d, respectively. Consistent behavior is observed at both temperatures across the systems. Hydrogels at pre-LCST temperatures exhibit a higher prevalence of hydrogen bonds with the solvent and fewer intramolecular hydrogen bonds. Conversely, post-LCST, these systems display a shift from polar interactions with the solvent to enhanced interchain interactions. This transformation is attributed to the partial expulsion of internal water, promoting interchain interactions.^27,28^

To delve deeper into these findings, the average water coordination number of both systems was investigated. Specifically, the average number of water oxygen atoms within the first hydration shell (*d* < 3.5 Å) of the NIPAAm residues was analyzed. Generally, a slight decrease in the coordination number of water is observed at 40 °C compared to values at 25 °C, as depicted in Figures 6a and S7. This result is consistent with earlier MD calculations^73^ and is supported by experimental findings like IR spectroscopy.^27,28^ Notably, the backbone atoms, C1 and C2, and C4 of the isopropyl group exhibit the lowest water coordination numbers due to steric hindrance, followed by those corresponding to the hydrophobic isopropyl group (C5 and C6). In contrast, the polar amide group’s oxygen and hydrogen atoms show the highest coordination numbers. The coordination values align with the two and one hydrogen bond assigned by Raman studies to the carbonyl and amine residues of the PNIPAM.^29^

**Figure 6:**
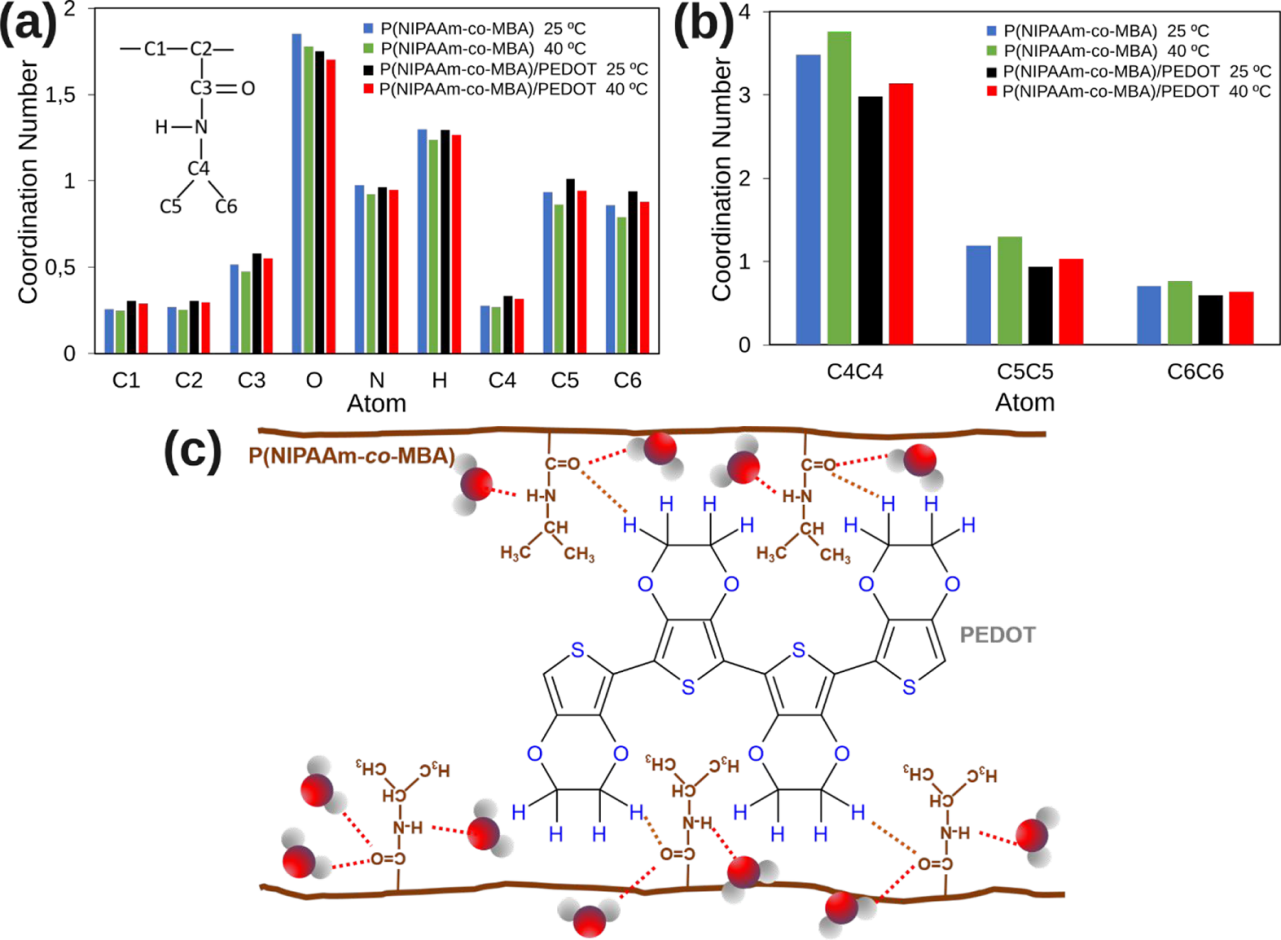
Coordination numbers of (a) water oxygen atoms in the first solvation sphere around each type of NIPAAm atoms, (b) carbon atoms between interchain isopropyl group; and (c) interaction scheme between P­(NIPAAm-*co*-MBA) and PEDOT.

The temperature’s impact is interesting, as the water coordination number decreases at post-LCST temperatures for all atoms, regardless of their polarity. This suggests a partial displacement of water out of the system, as expected. However, different behavior was observed when comparing systems with and without CP. The introduction of PEDOT chains within the polymeric network of PNIPAAm maintains a robust interaction with the carbonyl group (C=O) of the NIPAAm residue, as discussed in section 3.4 above. Consequently, water linked to the carbonyl group is displaced toward the hydrophobic zone of the NIPAM residue, diminishing its stability within the system. This phenomenon is substantiated by the reduction in the water coordination number of the carbonyl group’s oxygen and the increased water coordination in less polar atoms in the presence of PEDOT. Indeed, a close inspection of the interchain coordination number involving carbon atoms of the isopropyl group (C4–C4, C5–C5, C6–C6), as illustrated in Figure 6b, reveals an increment in the interchain coordination number of the isopropyl moiety when comparing pre- and post-LCST systems. This behavior is a clear indication that beyond the LCST point, the PNIPAAm hydrogel engages with fewer water molecules, concurrently enhancing interchain hydrophobic interactions among nonpolar isopropyl groups. It is noteworthy that the incorporation of PEDOT within the PNIPAAm polymer network contributes to a reduction in the hydrophobic interactions between different PNIPAAm chains, attributed to the screening effect of PEDOT. Essentially, CP facilitates water displacement, analogous to the effect observed at post-LCST temperatures. Figure 6c illustrates the mechanism of the PEDOT-induced shielding effect on the PNIPAM chains. However, unlike what happens with an increase in temperature, CP induces an increase in the SR. Furthermore, it results in a significant augmentation of system porosity, primarily attributed to the screening effect on hydrophilic interactions within the PNIPAAm chains. This phenomenon is less pronounced at temperatures beyond the LCST, as illustrated in Figure 2b.

On the other hand, at temperatures surpassing the LCST, hydrophobic interactions are predominant, and the CP screen effect on polar moieties of PNIPAAm further bolsters the hydrophobic collapse of PNIPAAm itself. This phenomenon manifests in an observed increase in hydrophobicity, as depicted by the experimental results of WCA measurements in Figure 3d.

### 3.6 Effect of the Environment on the Hydrophilic–Hydrophobic Transitions of PNIPAAm

Since the pioneering publication of Lin et al.,^27^ both IR spectroscopy and Raman spectroscopy^29^ have emerged as important tools for examining temperature-induced phase transitions within PNIPAAm systems, whether in aqueous environments or in the form of hydrogels. The alterations in internal structures during the phase transition, involving the uncoupling of water molecules from the amide residues of PNIPAAm, and the transformation of the environment toward increasingly hydrophobic states, have been clarified by discerning shifts in frequency and changes in absorption intensity within the amide and methyl bands of the isopropyl group of PNIPAAm.^25,74^ Undoubtedly, the microenvironmental composition impact is pivotal in shaping the observed behavior unveiled by IR or Raman spectroscopy.

To delve into the atomistic intricacies of intra- and intermolecular arrangements of thermosensitive hydrogel chains under temperature stimuli and in the presence of CP, a high-level spectroscopic theoretical investigation of PNIPAAm-*co*-MBA systems was conducted with and without PEDOT chains, implicitly accounting for environmental factors.

Starting with systems previously equilibrated through MD, ten snapshots were extracted for each one at 25 and 40 °C. A subsequent minimization at the hybrid quantum mechanics/molecular mechanics molecular dynamics (QM/MM MD) level was performed to derive the IR/Raman spectrum of the system. Figure 7 provides a detailed description of the quantum region defined for each system, thereby focusing the spectroscopic inquiry on two fused NIPAAm/MBA residues and the 3­(NIPAAm)/EDOT_2_ interaction set for the P­(NIPAAm-*co*-MBA) and P­(NIPAAm-*co*-MBA)/PEDOT systems, respectively.

**Figure 7:**
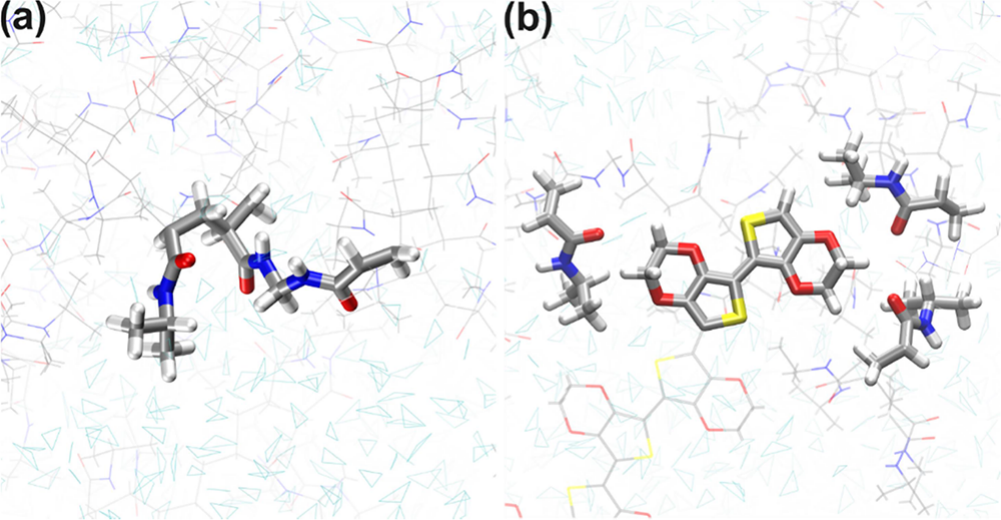
Detail on the quantum region of (a) P­(NIPAAm-*co*-MBA), and (b) P­(NIPAAm-*co*-MBA)/PEDOT systems. The quantum atoms are highlighted in licorice representation; meanwhile, the classic atoms are represented in lines. C atom (gray), N atom (blue), O atom (red) and H atom (white).

This data was utilized to train an artificial neural network (ANN) for detecting the normal modes of AmI, AmII, AmIII, and CH_3_ deformation of the isopropyl group of PNIPAAm, and the stretching vibration of C_α_=C_β_ in the five-membered ring of the EDOT residues. Following ANN training, notable accuracies were achieved, with 97.8% for AmI, 95.7% for AmII, and 87.5% for AmIII modes. Concurrently, the accuracies for CH_3_ deformation of the isopropyl group of PNIPAAm and the C_α_=C_β_ stretching vibration in the PEDOT five-membered ring both reached 100%. However, the results for AmIII were not included in this work due to their lower precision and inherent identification challenges.

To ensure robust statistical insights into the two systems under examination, a 6 ps hybrid QM/MM MD trajectory was generated at both 25 and 40 °C. Following trajectory generation, 25 snapshots were extracted per system, each obtained at the postequilibration within distinct solvation environments of the QM region. A total of 100 snapshots underwent processing, entailing QM/MM MD minimization and frequency calculations involving the previously defined QM region. The utilization of the two pretrained ANN models facilitated the swift extraction of information from the normal modes of the amide, the isopropyl group of PNIPAAm, and the thiophene ring from each derived spectrum.

Figure 8 delineates the statistical representation of AmI and AmII normal modes along with their Raman intensity for the P­(NIPAAm-*co*-MBA) hydrogel. The obtained results align perfectly with experimental observations. Specifically, the average frequency of AmI increased from 1636 to 1646 cm^–1^ during the PNIPAM’s hydrophobic collapse between 25 °C (pre-LCST) and 40 °C (post-LCST). This frequency shift is concomitant with an increase in intensity, in perfect agreement with the experimental data detailed in Table 2. Similarly, the average frequency of AmII experiences a marginal decrease with rising temperature, particularly around the LCST point, aligning closely with experimental observations.^25,29,74^

**Figure 8:**
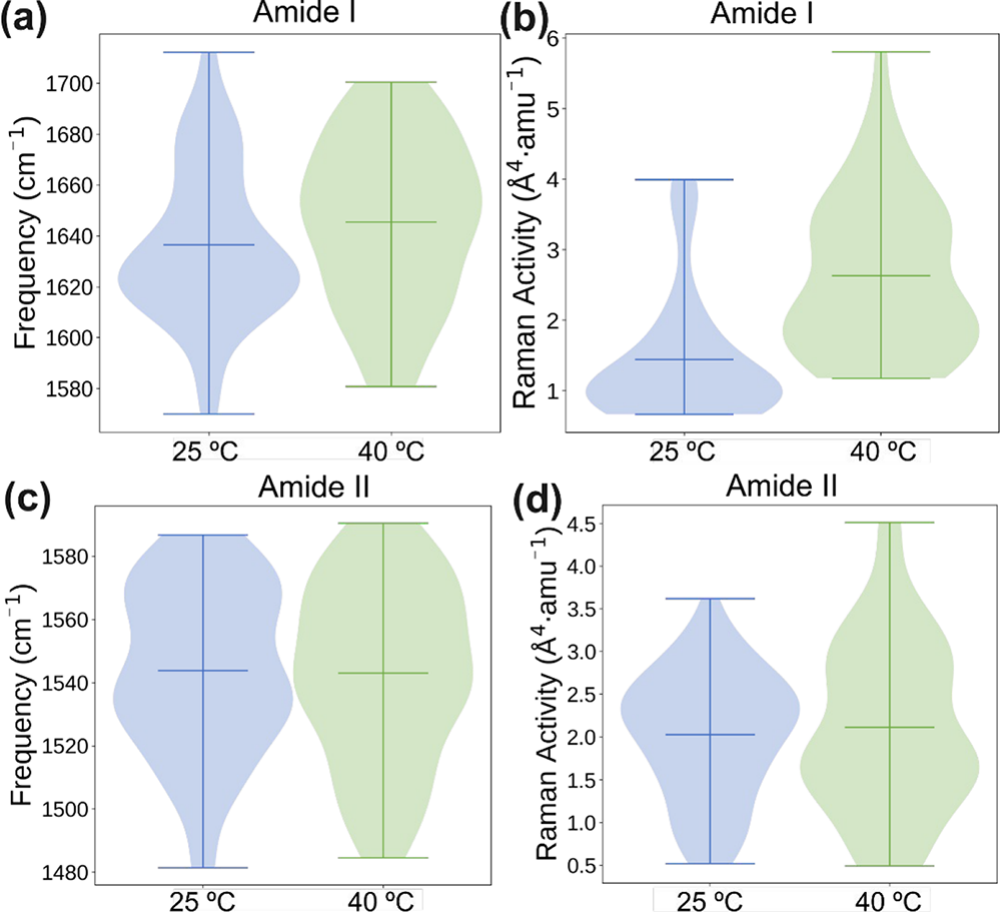
Statistical analysis on the AmI band: (a) frequency and (b) Raman scattering activity. AmII band: (c) frequency and (d) Raman scattering activity. Result obtained from QM/MM MD trajectories of P­(NIPAAm-*co*-MBA) at 25 and 40 °C

**Table 2:** Averaged Apparent Vibrational Frequencies (ν, in cm^–1^), Infrared Intensities (*I* ^IR^, in km mol^–1^), and Raman-Scattering Activities (*I* ^Ram^, in Å^4^ amu^–1^) for P­(NIPAAm-*co*-MBA) and P­(NIPAAm-*co*-MBA)/PEDOT at Different Temperatures (*T*, in °C)^a^

mode	*T*	ν	ν_exp_	*I* ^IR^	*I* ^Ram^
P(NIPAAm-co-MBA)					
amide I	25	1636 ± 31	1625^b^; 1624^c^	267 ± 71	1.4 ± 0.9
	40	1646 ± 33	1650^b^; 1654^c^	291 ± 119	2.6 ± 1.1
amide II	25	1544 ± 26	1561^d^	252 ± 60	2.0 ± 0.7
	40	1543 ± 28	1552^d^	246 ± 133	2.1 ± 1.0
P(NIPAAm-co-MBA)/PEDOT					
CH_3_ deformation	25	1451 ± 1	1456^e^	22 ± 0	0.8 ± 0.5
	40	1453 ± 2	1465^e^	14 ± 2	10.7 ± 1.9
C_α_=C_β_ stretching	25	1527 ± 5^f^	1438^e^	12 ± 17	2001 ± 267
	40	1526 ± 6^f^	1432^e^	8 ± 12	2819 ± 718

a Measures were taken from vibrational modes after QM/MM MD simulations.

b Ref 74.

c Ref 29.

d Ref 25.

e This work (deconvoluted peaks).

f Theoretical EDOT dimer frequency.

On the other hand, considering the P­(NIPAAm-*co*-MBA)/PEDOT system, the frequency ν_stCα=Cβ_ extracted from the theoretical spectrum (1527 cm^–1^) significantly deviates from the anticipated experimental value (1435 cm^–1^). The band assignment was verified by direct visual inspection of the normal vibration modes derived from the simulation. This discrepancy arises from employing the EDOT dimer within the QM region of the simulation instead of a more extensive oligomer to emulate the polymer. To address the high considerable computational demands associated with QM/MM MD simulations, a pragmatic decision was made to employ a minimalist QM region, enhancing the feasibility of the theoretical simulation.

To substantiate this rationale, theoretical spectra were generated using the GaussSum program,^75^ incorporating frequencies and Raman scattering activities of P­(NIPAAm-*co*-MBA)/PEDOT previously obtained. Raman intensities were computed with an excitation wavelength of 785 nm at corresponding pre- and post-LCST temperatures of 25 and 40 °C. Figure 9a depicts the theoretical Raman spectra of the P­(NIPAAm-*co*-MBA)/PEDOT system at 25 and 40 °C, and being compared with the theoretical spectra of the doped dimer (EDOT_2_^+1^:HSO_4_^–1^) and the doped hexamer (EDOT_6_^+3^:3HSO_4_^–1^) of PEDOT in vacuum (Figure 9b), all obtained at the same theoretical calculation level.

**Figure 9:**
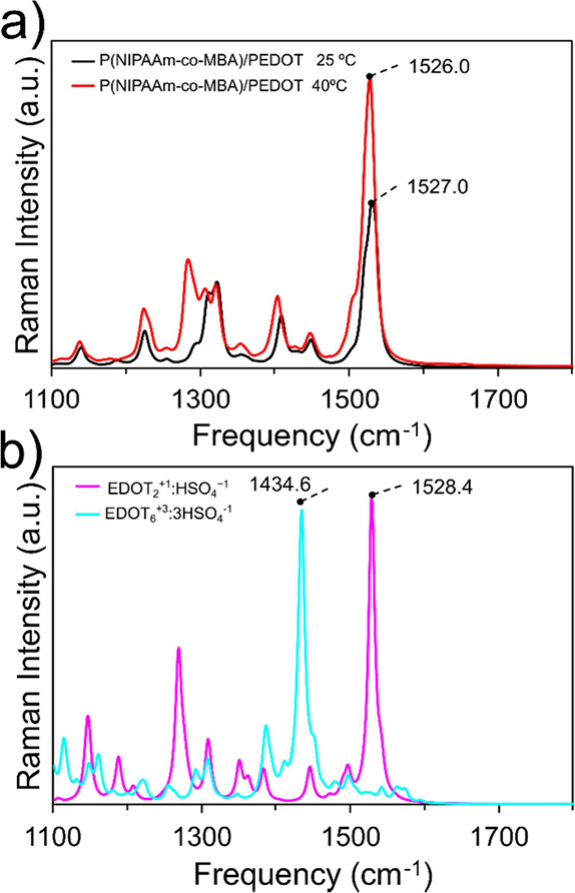
Theoretical Raman spectra of the (a) doped dimer (EDOT_2_^+1^:HSO_4_^–1^) and the hexamer (EDOT_6_^+3^:3HSO_4_^–1^) of PEDOT and (b) the P­(NIPAAm-*co*-MBA)/PEDOT system at 25 and 40 °C.

The alignment of the vibration frequency ν_stCα=Cβ_ of the doped dimer (EDOT_2_^+1^:HSO_4_^–1^) with values obtained from the QM/MM MD calculations is evident. Furthermore, it closely matches a recently acquired experimental Raman spectrum of a comparable EDOT dimer doped with HSO_4_^–1^ ions.^76^ Conversely, the doped hexamer system (EDOT_6_^+3^:3HSO_4_^–1^), known for reproducing representative Raman spectra of the polymer,^77^ yields a value of ν_stCα=Cβ_ = 1434.6 cm^–1^. This result concurs with our experimental measurements and is consistent with previous theoretical and experimental studies.^66,77,78^ The blue shift observed in the dimer, compared to those of the hexamer and the polymer, is primarily attributed to the predominantly benzoic structure exhibited by the mentioned system. Examination of the bond-length alternation patterns in all of the calculated systems (Figure S9) indicates that the hexamer predominantly adopts a quinoid structure at the center of the oligomer. In contrast, both the dimers in the vacuum and those within the hydrogel showcase a predominantly benzoic structure. Upon closer inspection of Figure 9, it is evident that the variations in Raman scattering activity within the ν_stCα=Cβ_ frequency band are more influenced by microenvironmental factors than by the amount of EDOT residues used in the oligomer simulation, contrary to what happens with the frequency value. Therefore, the theoretical ratio (ϕ = *I* ^Ram^_stCα=Cβ_/*I* ^Ram^_defCH3_) between the Raman intensities established for the dimer can be extrapolated to the polymer and compared with the experimental ϕ ratio reported above.

Deepening the analysis of the P­(NIPAAm-*co*-MBA)/PEDOT system, we observed that the peak centered around 1450 cm^–1^ originated from the nearly overlapping bands of the ν_defCH3_ of the isopropyl moiety of NIPAAm (1460 cm^–1^) and the ν_stCα=Cβ_ in the five-membered ring of PEDOT (1435 cm^–1^). This particular composed peak exhibited a noticeable shift of 20 cm^–1^ during the hydrophobic collapse process of PNIPAAm, elucidated by the alteration in the intensity of the superimposed absorption bands (see Section 3.3 for details).

A more detailed examination of the absorption peaks in the theoretical spectra corresponding to these bands substantiates the experimental hypothesis outlined above. In Figure 10a,b, the ν_defCH3_ in the isopropyl group of PNIPAAm experiences a slight increase, accompanied by a substantial enhancement in Raman scattering activity (∼1240%, as indicated in Table 2) when the system surpasses the LCST point. Conversely, ν_stCα=Cβ_ (depicted in Figure 10c,d) undergoes a marginal downward shift above the LCST, with a minimal increase in Raman scattering activity (∼41%, Table 2).

**Figure 10:**
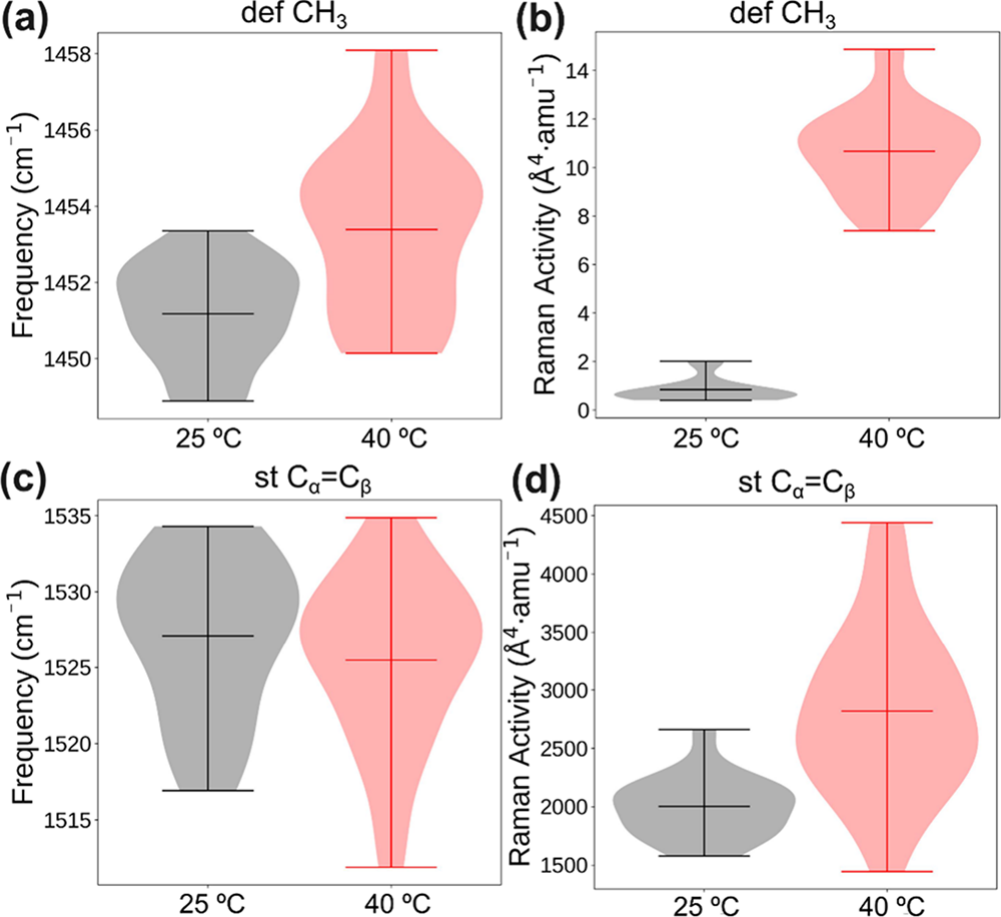
Statistical analysis on the CH_3_ deformation of the isopropyl group of the PNIPAAm band: (a) frequency and (b) Raman scattering activity. Stretching vibration of C_α_=C_β_ on the five-member ring of PEDOT: (c) frequency and (d) Raman scattering activity. Result obtained from QM/MM MD trajectories of P­(NIPAAm-*co*-MBA)/PEDOT at 25 and 40 °C.

Consequently, the ratio (ϕ = *I* ^Ram^_stCα=Cβ_/*I* ^Ram^_defCH3_) between the Raman intensities centered on both peaks diminishes from 2502 to 264 during the transition from the hydrophilic to the hydrophobic state of PNIPAAm. This change primarily stems from the significant increase in the Raman-scattering activities of ν_defCH3_. This observation aligns with the findings from the deconvolutional study of the corresponding band at the experimental level (see section 3.3).

## 4 Conclusions

The molecular interactions between a 3D structure of a NIPAAm hydrogel cross-linked with MBA and a conductive polymer (PEDOT NPs) were explored in this study by combining experimental and computational simulations.

An increase in SR was induced by the presence of PEDOT across all temperatures with a notably greater increment observed at pre-LCST values. Additionally, a two-fold increase in the hydrogel pore size and porosity (up to 79.4%) was noted upon the addition of CP. It also intensified hydrogel hydrophobicity, particularly posthydrophobic collapse. Temperature effects were investigated through Raman spectroscopy, unveiling a robust band in the 1400–1500 cm^–1^ region. A blue shift of 20 cm^–1^ indicated the hydrophobic collapse of PNIPAM above the LCST, showcasing PEDOT’s influence on modifying the microenvironment of NIPAM chains and existing interactions. High-level atomistic simulations validated these experimental findings, with theoretical Raman spectra confirming trends observed through the VPT. In addition, a DFT-level study highlighted the role of weak van der Waals forces in the NIPAAm/EDOT complex. Stable complexes exhibited a robust interaction between the C=O group of NIPAAM and the hydrogen of the dioxane ring of EDOT. The latter interaction is precisely responsible for the mild shielding of the NIPAAm residues. This shielding effect leads to a decrease in the number of hydrogen bonds with water, resulting in a dehydrating effect. Subsequently, water migrates toward the more hydrophobic regions of the NIPAM residue, as evidenced by an increase in the number of water molecules in the first coordination sphere of the hydrophobic residues. This effect is similar to that observed in the hydrophilic–hydrophobic state transition through the LCST point with increasing temperature of the hydrogel.

These findings underscore PEDOT’s significant influence on the hydrophilic–hydrophobic transition and the associated structural features of the supporting polymeric systems. Understanding this impact is crucial for the systematic development and fine-tuning of the behavior of new CPHs, ensuring better adaptation to their intended final applications. Moreover, the application of hybrid simulation methods in unraveling coil-to-globule transition pathways has proven to be a promising approach for studying complex stimuli-responsive polymer systems.

## Supplementary Material



## Notes

Additional detail on the cluster analysis, structures of NIPAAm-EDOT complexes, PEDOT NP size distribution, and results on the model system analysis in this study (PDF)
