# Supplementary material for: Exploring the Effects and Interactions of Conducting Polymers in the Volume Phase Transition of Thermosensitive Conducting Hydrogels
Source: Chem Mater. 2024 Apr 17;36(9):4688–702. doi: 10.1021/acs.chemmater.4c00433 (PMC12308728; doi:10.1021/acs.chemmater.4c00433)
Supplement: Supplementary file 1 [file cm4c00433_si_001.pdf]

Supporting Information

Exploring the Effects and Interactions of Conducting Polymers in the Volume Phase Transition of Thermosensitive Conducting Hydrogels

David Naranjo,[a,b] *Sofia Paulo-Mirasol,*[a,b] *Sonia Lanzalaco,*[a,b] *Haoyuan Quan,*[a] *Elaine Armelin,*[a,b] *José García-Torres,*[b,c] *and Juan Torras*[a,b*]

[a] IMEM-BRT Group, Departament d'Enginyeria Química, EEBE, Universitat Politècnica de Catalunya, C/ Eduard Maristany, 10-14, Ed. I, 2nd floor, 08019, Barcelona, Spain.

[b] Barcelona Research Center in Multiscale Science and Engineering, EEBE, Universitat Politècnica de Catalunya, C/ Eduard Maristany, 10-14, basement S-1, 08019, Barcelona, Spain.

[c] Biomaterials, Biomechanics and Tissue Engineering Group, Department of Materials Science and Engineering and Research Center for Biomedical Engineering, Universitat Politècnica de Catalunya (UPC), 08019 Barcelona, Spain.

*Corresponding author: joan.torras@upc.edu

Table S1. Distance between NIPAAm and EDOT centers of mass and Binding energies of the NIPAAm-EDOT complexes

Cluster	BE_{CP} (kcal mol^{-1})	$d_{r\text{-}r}$ (Å)
A	-8.26 ± 0.31	3.64 ± 0.37
B	-7.85 ± 0.38	4.45 ± 0.26
C	-7.65 ± 0.53	3.85 ± 0.29
D	-6.52 ± 0.29	4.77 ± 0.42
E	-5.88 ± 0.59	3.93 ± 0.51
F	-5.87 ± 0.56	4.15 ± 0.27
G	-5.69 ± 0.43	5.83 ± 0.21
H	-5.25 ± 0.61	6.11 ± 0.53
I	-4.44 ± 0.31	5.37 ± 0.32
J	-3.11 ± 0.53	6.04 ± 0.28
K	-1.68 ± 0.67	7.45 ± 0.66
L	-1.63 ± 0.16	7.60 ± 0.24
M	-0.35 ± 0.00	9.51 ± 0.00
N	-0.01 ± 0.00	13.55 ± 0.00
O	0.00 ± 0.00	10.80 ± 0.00

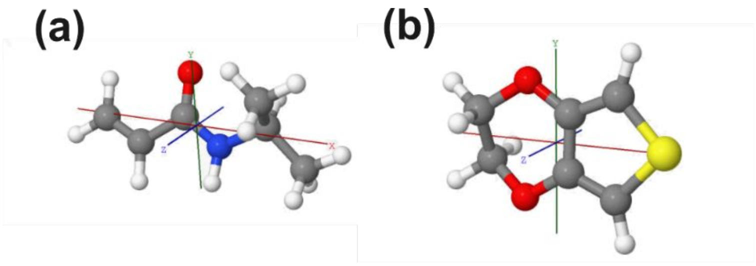

Figure S1. Optimized structures of a) NIPAAm, and b) EDOT monomers.

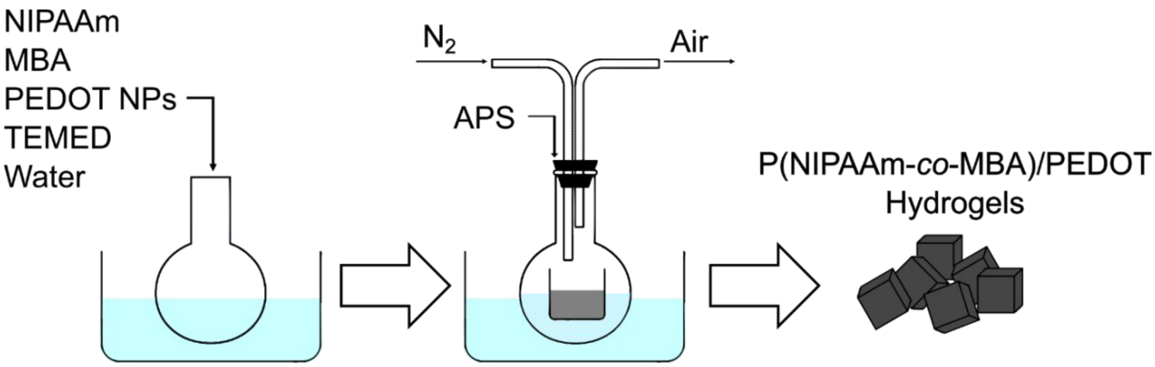

Figure S2. Scheme of P(NIPAAm-*co*-MBA)/PEDOT hydrogel fabrication.

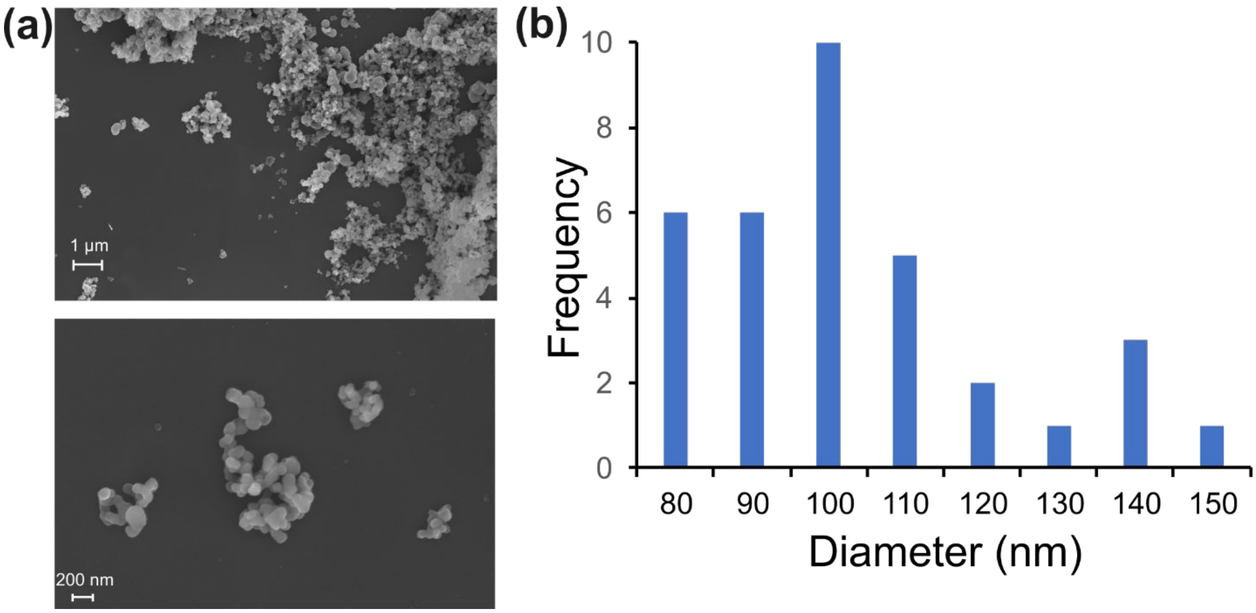

Figure S3. (a) SEM micrograph with 5.1 kX and 22.9 kX of magnification and (b) size particle distribution of PEDOT NPs.

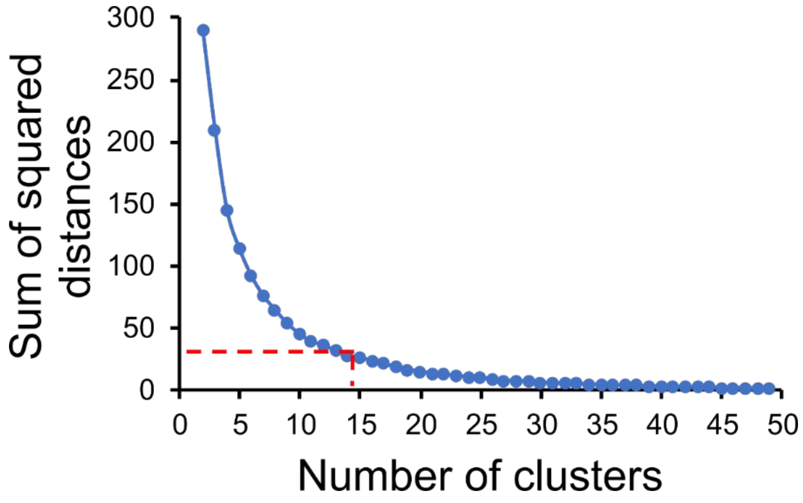

Figure S4. Sum of squared distances of the geometric and energetic variables of the system NIPAM-EDOT as a function of the number of clusters.

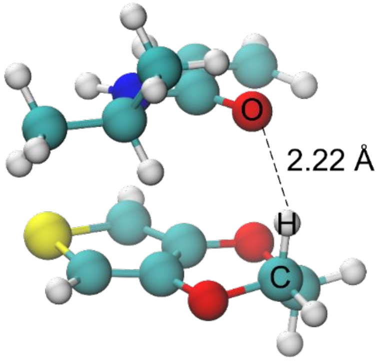

Figure S5. Illustration of the most stable Structure of the NIPAAm/EDOT complex. Representative structure of cluster A.

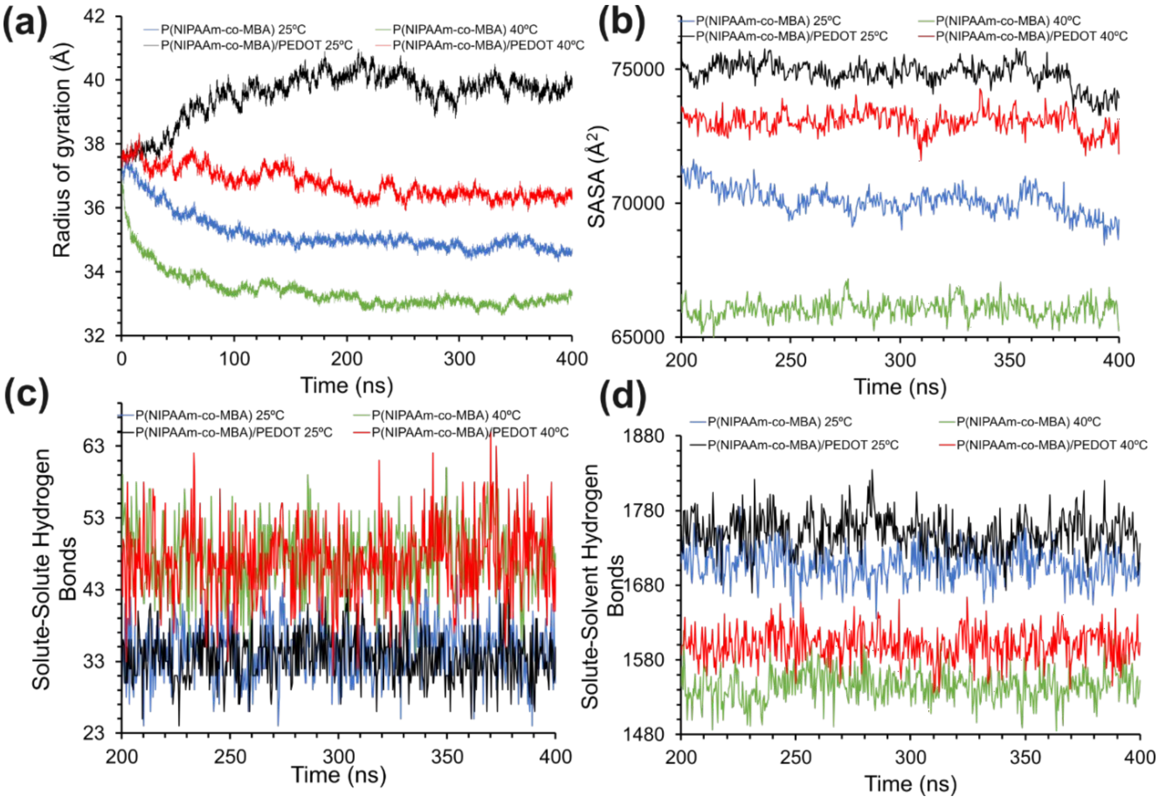

Figure S6. (a) Radius of gyration, (b) solvent accessible surface area, (c) number of solute-solute hydrogen bonds and (d) number of solute-solvent hydrogen bonds of P(NIPAAm-*co*-MBA) and P(NIPAAm-*co*-MBA)/PEDOT at 25 °C and 40 °C.

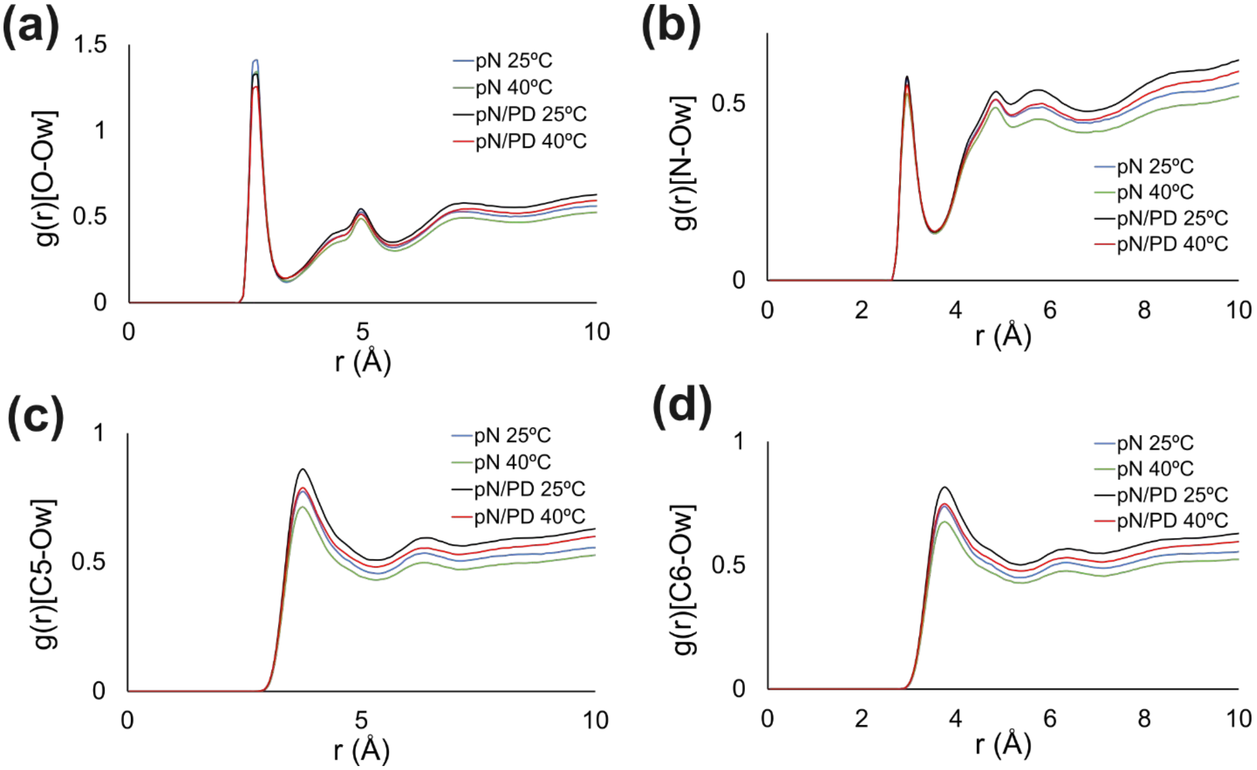

Figure S7. Radial distribution function between a) carbonyl oxygen, b) nitrogen, c) methyl carbon (C5), and d) methyl carbon (C6) atoms of PNIPAAm residues to water oxygen atom (Ow). Data derived from classical MD trajectories of P(NIPAAm-*co*-MBA) and P(NIPAAm-*co*-MBA)/PEDOT at 25 °C and 40 °C.

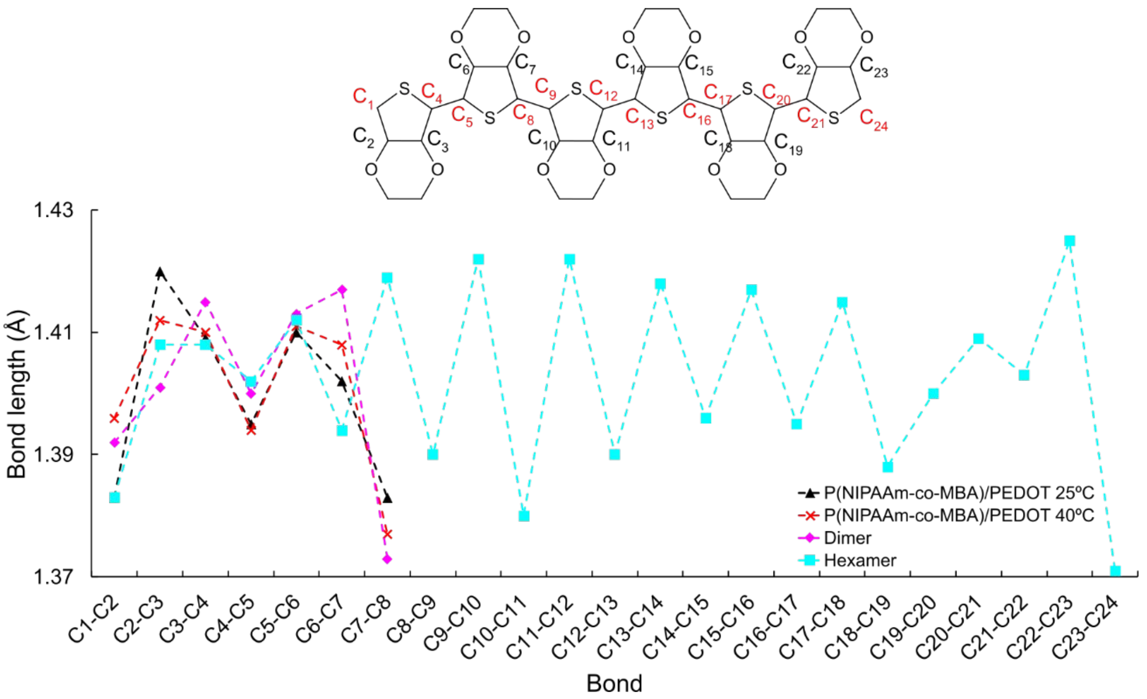

Figure S8. Bond-length alternation patterns of the CC bond along the conjugated π-system of the dimer and hexamer at the vacuum, and the simulated P(NIPAAm-*co*-MBA)/PEDOT system at both temperatures. The data were taken from the structures optimized at the DFT level and from the QM/MM MD calculations, respectively.
